# Museotherapy as a method: an investigation into the emotional experiences of Chinese youth visitors

**DOI:** 10.3389/fpsyg.2025.1716551

**Published:** 2026-01-16

**Authors:** Siyi Wang, Xuedan Gong, Wenting Lian

**Affiliations:** 1 Department of Archaeology and Museology, Shanghai University, Shanghai, China; 2 Aurora Museum, Shanghai, China

**Keywords:** China, emotional experience, museotherapy, university students, visitor studies

## Abstract

Rising mental health challenges among Chinese youth necessitate new intervention models. This study explores the mechanisms of museotherapy workshops in regulating emotions and fostering self-discovery among university students, aiming to validate museums as therapeutic environments. Structured workshops were conducted at the Shanghai University Museum involving 81 students aged 17–22. By integrating Appraisal Theory and narrative therapy, the study employed a mixed-methods approach—utilizing text sentiment analysis (SPSSAU) and facial expression coding (FAST)—to evaluate participant responses to specific “healing objects.” Text analysis revealed a predominantly positive emotional shift (83.33% positive), identifying museum objects as the primary emotional triggers (accounting for 69.5% of perception statements). Furthermore, video analysis identified a characteristic “W-shaped” emotion curve, mapping the participant journey from initial engagement to deep reflection and distinct positive resolution. These findings validate museotherapy as an effective method for anxiety alleviation, operating through mechanisms of sensory stimulation, psychological resonance, and dialogue. Consequently, this research offers a robust framework for evaluating the emotional impact of museum interventions.

## 1 Introduction

As the pressure in modern society grows, young people’s mental health problems are becoming increasingly prominent, and there is a trend toward younger age groups. According to the “China Mental and Psychological Health Report” (China National Narcotic Drugs Association, 2023), the depression risk among individuals aged 18–24 reaches as high as 24.1%, ranking first among all age groups. Among university students, the risk of mild anxiety is 38.26%, mild depression is 16.54%, and severe depression is 4.94% (China National Narcotic Drugs Association, 2023). Since the COVID-19 pandemic, a substantial increase in the prevalence of affective disorders, for example, a 27% increase in depressive disorders and a 25% increase in anxiety disorders (Santomauro et al., 2021), as well as other mental health burdens, such as posttraumatic stress disorder (e.g., Wathelet et al., 2021), insomnia and distress (Wu et al., 2021), and chronic pain (Clauw et al., 2020), has been reported (Daniali et al., 2023). Three-quarters of mental health problems emerge before the age of 25, but people aged 16–24 years constitute the age group that is least likely to seek help (Andrews et al., 2001; Olfson and Klerman, 1992). The mental health of students has increasingly become a significant concern for major universities (Li et al., 2025). As the focus on student mental health gains momentum, the unique value and potential of museums, particularly university museums as campus-based cultural institutions, in responding to such issues are becoming increasingly evident and demanded.

For centuries, individuals have sought museums, including outdoor spaces such as gardens, for solace and rejuvenation. These institutions were seen as a way to escape, for a period of time, the stressors of daily life (Ghadim and Daugherty, 2021). Ongoing research has confirmed that museums are categorized as restorative environments (Kaplan, 1995), and Deane et al. (2000) argue that the environment and space of museums have transformed into more social and inclusive platforms, placing greater emphasis on public education and services, and gradually emerging as a popular space for art therapy. In current healing practices, the use of museum objects is categorized into four primary approaches: verbal exchange, kinetic experiences and activities, connecting by making in galleries, and multisensory experiences (Dejkameh et al., 2018). Thus, museums have a unique ability to alleviate mental health challenges (Clow and Fredhoi, 2006; Sone et al., 2007).

Museotherapy (also known as museum therapy or object-based therapy) is a form of therapeutic intervention that uses encounters with museum objects and collections—such as artworks, historical artifacts, scientific specimens, or cultural items—within a museum or cultural setting to promote psychological, emotional, and social well-being. It is developed through the intervention and fusion of art therapy (Bondil and Legari, 2022; Chatterjee et al., 2009; Cull and Cull, 2022; Ioannides, 2016; Mangione, 2018). Early museum art therapy activities were mostly auxiliary treatments for physiological therapy (Wang et al., 2025). In 2018, the American Association of Museums emphasized 10 areas in which museums contributed to health care (American Alliance of Museums, 2013). The activities are often conducted for individuals suffering from psychological issues, mental disorders, cancer, anorexia, bulimia, post-surgery recovery, children with autism, and elderly people with age-related conditions (Table 1).

**Table 1:** Museum-based art therapy (MBAT) population(s) served.

Groups served	Example museum	References
Adolescents aged 13–17 years with a diagnosis of high-functioning autism (HFA)	Florida State University Diagnosis of high Museum of Fine Arts (MoFA)	Hartman (2019)
Young adults aged 18–25 years with complex mental health difficulties	Museum of Gloucester and Gloucester Life Museum	Coles et al. (2019)
Visually impaired persons (VIP)	Queens Museum Please Touch 1983 Program and Open Studio Programs	Dejkameh and Shipps (2019)
Individuals with dementia and Alzheimer’s disease and caregivers	Art *Access* Queens Museum Creative Imagination Project	Dejkameh and Shipps (2019)
Older adults with memory loss, diabetes, stroke, Parkinson’s disease, and other chronic illnesses, as well as family members	The Creative Aging Program at The Phillips Collection, Senior Center and Wellness and Arts Center George Washington University Art Therapy Program	Rosenblatt (2014)
People with eating disorders	Montreal Museum of Fine Arts (MMFA) and the Douglas Mental Health Institute Department	Baddeley et al. (2017)
Cancer patients	McMichael Canadian Art Collection and Bayview Cancer Support Network, Kleinburg, Ontario	Deane et al. (2000)
Refugees from Syria and Iran	National Museum of Contemporary Art, Athens (EMST), and UN Refugee Agency	Ioannides (2016)
Artists aged 5–19 years with PTSD and other mental health issues associated with trauma	Museum of City of New York and NYU Child Study Center	Goodman and Fahnestock (2002)

**Figure 1:**
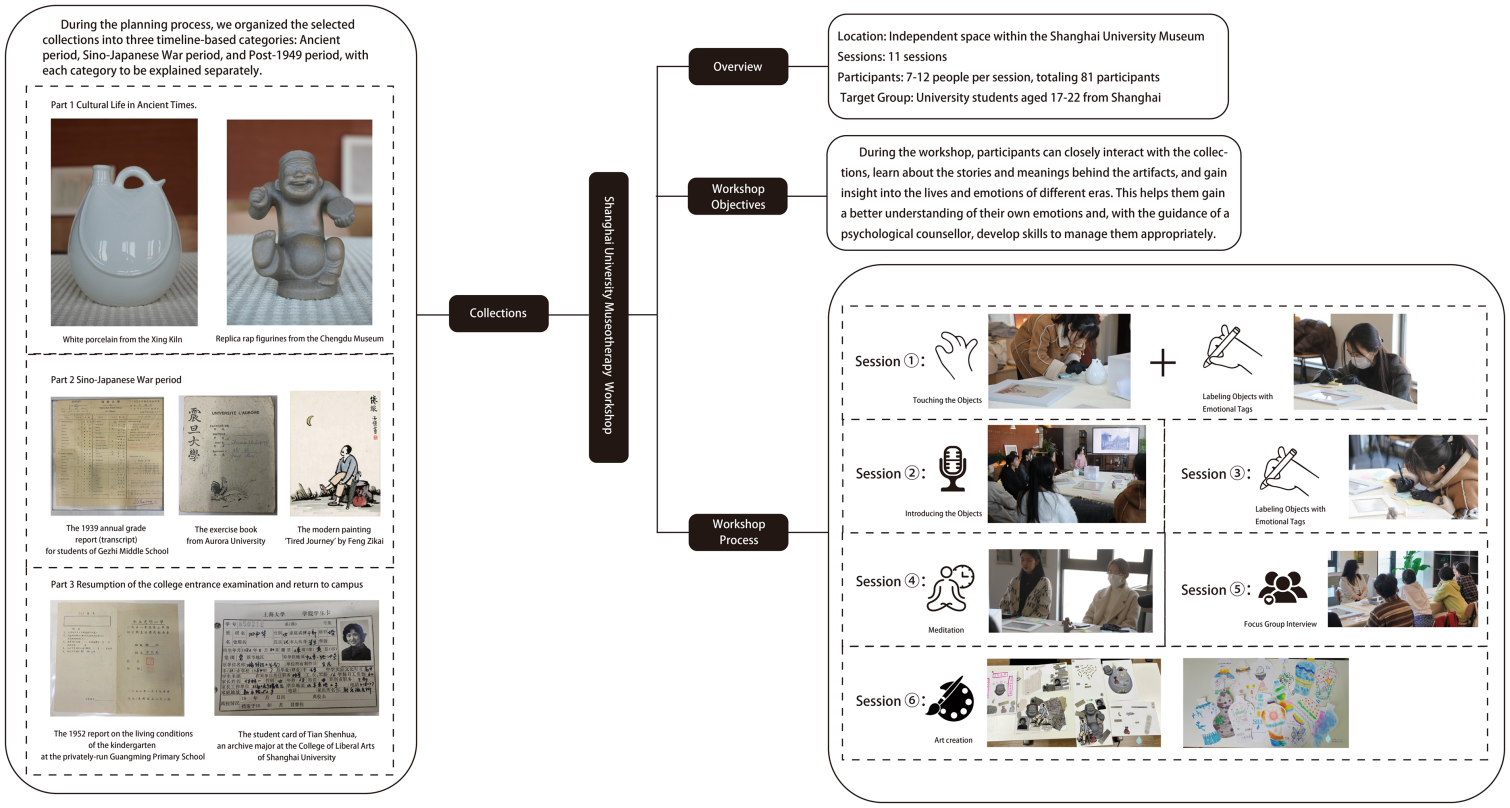
Workflow of the museotherapy workshops at the Shanghai University Museum.

In this context, museums may have additional functions, other than preserving and displaying cultural heritage. They may operate as centers of emotional experience that tap into their inherent potential for psychological healing. Thus, in 2023, we held 11 museotherapy workshops (Figure 1) at the Shanghai University Museum for university students aged 17–22 years. In this study, using videos recorded during the museotherapy workshop, along with the SPSSAU text emotion analysis tool,^1^ the expressions, behaviors, and interview dialogues of young participants were thoroughly analyzed through the methods of narrative-based dialogue framework and emotional evaluation. This approach examined their engagement in the workshop, offering valuable insights into the supportive effects of museotherapy workshops on young visitors.

## 2 Museum visitors’ emotional experience and analysis methods

The “museum fatigue” studies led by Robinson (1928) and Melton (1972) focused on examining the impact of museum environments on visitors’ behaviors. These studies significantly contributed to the development of observation as a key research method in museology. Meanwhile, the interdisciplinary integration of fields such as marketing and psychology introduced a broader range of research methods into visitor studies (Ceccarelli et al., 2024; Ferilli et al., 2016; Kılıçarslan et al., 2024; Pearce, 2009; Wells, 2014). This shift helped transition the focus of visitor research from solely “serving the museum” to placing greater emphasis on the inner experiences and emotional responses of individuals. The Museum Experience (Dierking and Falk, 1992) highlights the pivotal role of context in shaping learning, particularly within and through museums, which encompasses the totality of the experience, from the moment the thought occurs to someone that visiting a museum might be a good idea, through the visit itself, to the recollection of the experience days, weeks, and even years later (Falk and Dierking, 2016). Packer and Ballantyne (2016) define the visitor experience as a “subjective response,” emphasizing that it emerges from the dynamic interaction between an individual’s internal and external environments. From this perspective, “experience” refers to a person’s mood and internal changes, which involve personal cognitive, esthetic, emotional, and even spiritual levels. The visitor experience is a significant focus of visitor studies; however, within the complex concept of “experience,” the educational role of museums has traditionally been emphasized. Consequently, visitor studies have often prioritized cognitive aspects while overlooking emotional dimensions. Previous studies on museum visitors’ emotions have primarily explored various aspects, including the emotional factors influencing visitors’ learning experiences within museums (Anderson, 1989), the motivations driving individuals to visit museums (Gregory and Witcomb, 2007; Smith, 2020; Smith and Campbell, 2016; Watson, 2015), visitors’ emotional engagement, connection, and resulting behavioral outcomes (such as word-of-mouth, donation intentions, and revisit behavior) (Gregory and Witcomb, 2007; Smith, 2020; Smith and Campbell, 2016; Watson, 2015, 2016), as well as emotional experiences elicited through sensory activities (De Jong, 2018; Gregory and Witcomb, 2007; Tolia-Kelly et al., 2016; Witcomb, 2014). An increasing body of research highlights the positive psychological benefits associated with the experience of visiting museums (Ander et al., 2011; Chatterjee and Camic, 2015; Fujiwara, 2013). In terms of research methods, scholars have utilized self-report questionnaires, interviews, observation, and tracking without intervention (Serenko and Turel, 2019) to gather data from museum visitors and analyze their experiences (Binnie, 2010; Luebke et al., 2016; Reed, 2018), with the aim of investigating the emotional impact of museum activities.

In summary, this study employs a qualitative approach, utilizing semi-structured interviews and systematic observation, to analyze the emotional dynamics of young visitors participating in museotherapy workshops. To delve deeper into the mechanisms underlying emotions and emotional changes, appraisal theories of emotion are introduced to examine the emotional experiences of young visitors. Furthermore, as it was not feasible to observe each visitor directly without intervention, the entire process was video recorded. Visitors’ facial expressions and body movements were analyzed through the recordings, and this approach was combined with an examination of dialogue content to comprehensively deconstruct the process of emotional changes and the factors influencing them.

Text analysis (textual data) aimed to answer: “Which touchpoints in the museotherapy workshop triggered emotions?” and “What specific emotions were triggered?” It revealed the content of emotions, their triggers (e.g., specific artifacts), and the participants’ reflection on and narrative reconstruction of their own emotions. Video analysis (nonverbal data) aimed to answer: “What was the immediate intensity and dynamic change of the emotions?” It captured more automatic emotional responses from participants that were either unrecorded in speech or even unnoticed by themselves, thereby further delineating the “W-shaped curve” of emotional fluctuation throughout the entire workshop process.

When text analysis indicated that a particular session (e.g., interaction with a certain artifact) elicited strong positive emotional expressions, we would look for corresponding nonverbal evidence (e.g., smiles, relaxed posture) in the video analysis. For instance, when a participant described a notebook as evoking nostalgic happiness, the video captured them laughing heartily. This consistency significantly enhanced the validity of the conclusion that “artifacts can elicit positive emotions.” Conversely, when textual data were limited or emotional expressions were neutral, nonverbal data provided additional insight. For example, a participant might have spoken very little. Still, their focused expression during the art creation session and their forward-leaning posture during activities corroborated, on a nonverbal level, their state of engagement, enriching our understanding of the “neutral-to-positive” emotional spectrum.

## 3 Stimulation-resonance-dialogue: text analysis results

The emotional changes experienced by visitors during the workshop are dynamic and influenced by various factors, including the environment, object stimulation, and individual personal experiences. To gain deeper insights into visitors’ perspectives, focus group interviews were conducted in the form of dialogue during workshops led by a psychological counselor, which were conducted in groups of 7–8 participants. To enhance emotional connection among visitors during each session, we assigned individuals of similar ages to the same session. For instance, the first group consisted entirely of first- and second-year undergraduates, while the second group comprised fourth-year students. The focus group interviews employed unstructured questioning and adopted a conversational approach to help participants lower their psychological guard and feel at ease. The sessions are structured into the following sections: Firstly, participants are encouraged to recall the objects that made the deepest impression on them and share their emotional responses. Secondly, they are guided to reflect on the similarities between themselves and the historical figures associated with the objects, as well as to discuss any recent challenges they have faced in their own lives. Thirdly, the discussion delves deeper into the underlying reasons and emotional journeys behind their feelings, fostering an environment where other participants can offer suggestions or share their resonances. Participants were recruited through voluntary registration. As the activity was not designed as clinical research, we employed the Trait Anxiety Inventory (TAI) as a pre-test measurement tool (Spielberger, 1989) and established an exclusion criterion for participants scoring above 65. The TAI measures anxiety on a scale ranging from 20 (minimal anxiety) to 80 (maximal anxiety). It does not provide fixed cutoffs for “normal” or “abnormal” states but serves as a quantifiable indicator for comparing anxiety levels across individuals or groups. Generally, higher scores reflect more severe anxiety. Consequently, we determined the exclusion threshold based on both scale interpretation guidelines and the actual score distribution observed during registration. This empirical distribution—where participant scores ranged from 26 to 73—further validated the appropriateness of our predetermined exclusion criterion. Ultimately, we obtained valid interview responses from 81 participants, all aged between 17 and 22. The gender distribution of visitors varied significantly, with men comprising approximately 20% of the total.

### 3.1 Text sentiment analysis and emotional triggers

Text sentiment analysis involves extracting the emotions conveyed within textual content. With the development of social networks, it plays a significant role in decision-making and response to online public opinion (Wankhade et al., 2022). This process involves analyzing, processing, summarizing, and reasoning about subjective texts with emotional connotations. For example, the words “like” and “dislike” can appear in the same or similar context, such as I love reading books and I dislike reading books. By merely looking at word co-occurrences, we would learn similar vector representations of “like” and “dislike,” as these have similar lexical behavior. From a sentiment perspective, however, such vector representations should be very different, as they convey opposite polarity (Li et al., 2017). Thus, as this study progressed, the analysis gradually shifted from simple emotional words to more complex emotional sentences and emotional discourses.

Firstly, to ensure the analysis focused on the target research subjects, we removed all dialogue from the host (the psychological counselor) from the original interview transcripts, retaining only the participants’ quotations as the analysis corpus. After preprocessing, the final cleaned participant text used for analysis totaled 12,923 words and 180 segments. This study utilized the SPSSAU online platform^2^ for text sentiment analysis. The platform’s sentiment lexicon is composed of a combination of sentiment dictionaries from sources including BostonNLP, National Taiwan University, Tsinghua University, and HowNet, totaling 130,000 words.^3^

Secondly, within the text sentiment analysis module, we selected the “line” as the basic unit of analysis. This means the system treated each participant’s independent statement or response as a single unit for sentiment scoring. Its processing logic is as follows: first, it automatically segments the text using a built-in dictionary and identifies sentiment keywords; then, it calculates the intensity of sentiment words in the sentence via an algorithm, taking into account modifiers such as negation words and degree adverbs, and finally computes a comprehensive sentiment tendency score for the statement. Referring to the official SPSSAU explanation, the sentiment scores calculated by the platform are divided into four sentiment categories, specifically defined as follows: Strongly Negative: score range [−1, −1/3). Indicates the statement contains strong negative emotions. Moderately Negative: score range [−1/3, 0). Indicates the statement contains moderate or neutral negative emotions. Moderately Positive: score range [0, 1/3). Indicates the statement contains moderate or neutral positive emotions. Strongly Positive: score range [1/3, 1]. Indicates the statement contains strong positive emotions. If a word is not found in the sentiment dictionary, the prompt “Word not in sentiment dictionary” appears; in all other cases, a sentiment score is provided.^4^

After analysis via the above process, the platform ultimately performed sentiment scoring and classification on 180 independent statements. The statistical results were: 24 strongly negative, 6 moderately negative, 13 moderately positive, and 137 strongly positive statements. The combined total of strongly and moderately positive statements was 150, accounting for 83.33% of the total analyzed statements. This indicates that the majority of visitors who attended the workshop exhibited relatively positive emotional tendencies during the activity. The remaining 17% of moderately negative utterances primarily centered on participants’ recent personal struggles—such as difficulties in reducing phone use, dissatisfaction with their academic major, or coping with a recent breakup. These expressions functioned as a constructive emotional outlet during the session.

**Figure 2:**
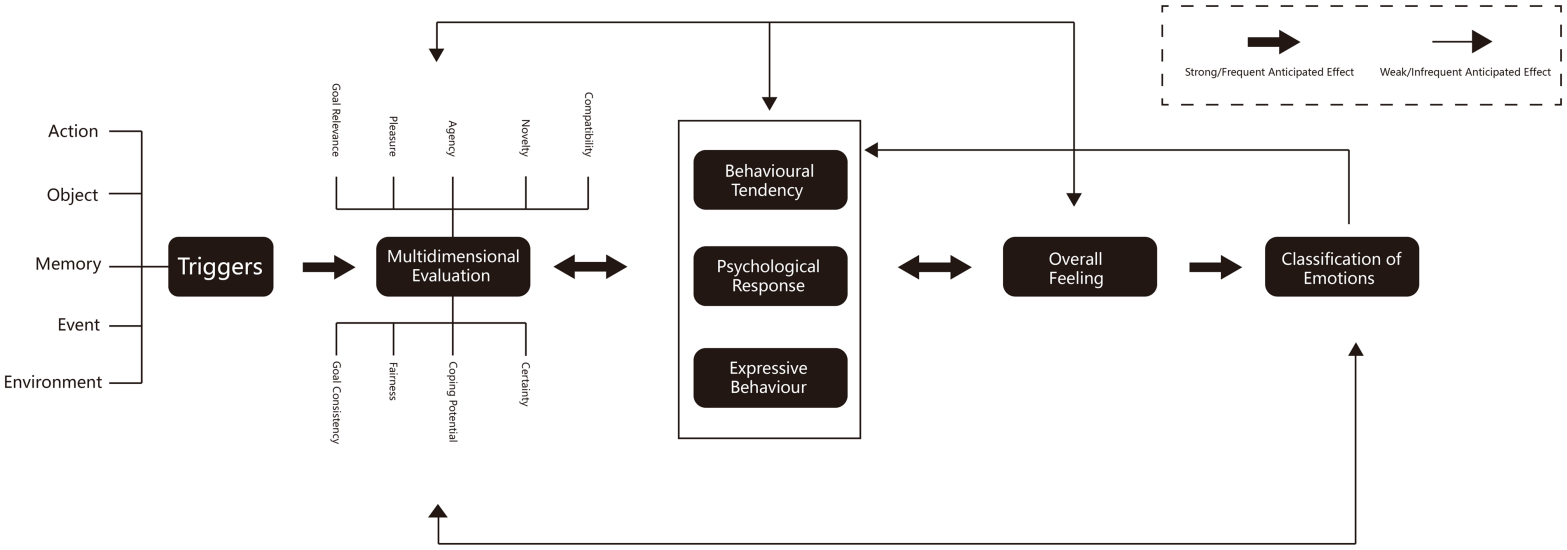
Framework of the appraisal theories of emotion.

Conscious emotional experience is a consequence of emotional evaluation (LeDoux, 2022). According to the appraisal theories of emotion, which is an emotion research method in psychology, appraisal values are integrated in a pattern, perhaps linked to some core relational theme (e.g., danger, loss), which determines the specific emotion that occurs (e.g., fear, sadness) (Ellsworth, 2024). Once this is determined, the other components that belong to the emotion are activated. The transition from the core relational theme to the other components may even be mediated by an affect program (i.e., a dedicated brain circuit for emotion) (Moors, 2014). Thus, based on appraisal theories of emotion, the authors treated each emotional expression from the visitors as an emotional appraisal process, working backwards from the outcomes to identify the stimuli, to explore the visitor’s emotional experience process (Figure 2).

The author found that the resulting texts could be categorized into three types (Table 2).

**Table 2:** Proportion of positive emotions for the three types of text.

Text generation	Text categorization	Amount of text (sentences)	Proportion of positive emotions
Collection, meditation, and creation activities	Visitor perception of the activity	82	91%
Focus group interviews	Self-reported anxiety	30	87%
	Anxiety countermeasures and trouble advice	69	74%

Eighty-two sentences documented visitors’ experiences and insights from the activities. This category primarily encompasses feedback on the process and subjective gains from participating in workshop sessions such as collection, meditation, and creation. The proportion of positive emotions here was the highest, at about 91%.

30 sentences comprised visitors’ self-reports on their anxiety. These texts were direct accounts where individuals described their personal feelings and states of anxiety. Notably, positive emotions still accounted for approximately 87% within this category.

69 sentences were visitors’ suggestions for alleviating their own current anxiety or for addressing others’ troubles, with positive emotions making up approximately 74%.

The analysis of the interview transcripts corroborated this finding, revealing that visitors’ descriptions of their feelings during the workshop were not only the most frequent type of expression but also the primary source of the positive emotions identified. The authors sorted the emotional triggers in the results (Table 3) and classified them. Among the 82 visitor perceptions, 57 stimuli came from the objects and the stories behind them, 21 came from art creations, and the remaining 4 were from up-close encounters with objects, the overall workshop experience, meditation and sharing by others. The objects and their connotations were the most arousing positive emotions in the visitors’ perceptions.

**Table 3:** Example of the categorization of visitor perception triggers.

Text	Trigger in the workshop	Classification of triggers
Oh, it is his transcript from Gezhi Middle School, right? Because right after he graduated, he was immediately thrown into a time of war. It is honestly such a pity—these students could have had such bright futures, but because of the times, their lives did not get a chance to improve.	Transcript	Objects and their connotation
When I saw this piece of white porcelain, I felt it was so exquisite and translucent. The first thought that came to my mind was: What did people in ancient times use it for?	White porcelain	
The rap figurine. I feel it is a bit scary	Rap figurine	
Seeing a transcript feels particularly sensitive; it can easily cause anxiety.	Transcript	
I feel like people back then probably also got distracted and doodled something here and there. I think that is kind of similar to how it is now.	Aurora University exercise book	
I guess it might have been used to hold water, but honestly, if it were me, I would not have the heart to use it for that—it feels too precious. So I’m really curious, would people in ancient times actually use something so valuable just for holding water?	White porcelain	
I think the student card left a deep impression on me. It is a thing that is relatively close to us now. Everyone has their own student card, which records a lot of information. Then, it can be found that she was getting married after working. I think it is very rare for me to go to university again in the future.	Tian Shenhua student card	
I think this painting is more impressive. The artist is painting himself, but I feel that he also wants to have a conversation with us through this painting, and I have a little empathy. When I first looked at this painting, I thought the person inside was relaxing, but after listening to the explanation just now, I think that he may actually be a little anxious, because he is looking at the moon and he does not know what he is doing. What he is thinking about, he may be thinking about his own goal, or his hometown. In his case, his emotion is not just as simple as relaxation.	“Tired Journey”	
I drew him listening to music. I also like it more, for example, shuffle play. During shuffle play, what you think of through the music and what the music wants to express have two meanings, which is very interesting. For the person in the painting, it is good for him to sit here and listen to the music. I hope he can be happier.	The secondary creation of “Tired Journey”	Creation

### 3.2 Connecting objects with youth’s emotions

The literature has cited very few reasons or criteria for the selection of healing objects in museums, and descriptions of the selection process in specific case studies have been overly general (Cutler, 2022; Ghadim and Daugherty, 2021; Watson et al., 2021). Therefore, this study integrates relevant core literature from psychology, neuroscience, medicine, and anthropology to propose a system of elements for museum healing objects, which is categorized into two dimensions: physical and psychological. The physical dimensions are subdivided into visual (Table 4) and tactile (Table 5), and the psychological dimensions are subdivided into religion (Table 6), myth (Table 7), and stories (Tables 8, 9).

**Table 4:** List of visual categories.

Category	Element/variable	References	Collections from museums
Visual color	Blue	Kaiser (1984) Valdez and Mehrabian (1994) Li and Zhang (2009)	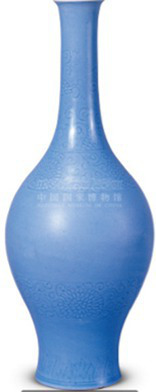 Qing Dynasty Sky-blue-glazed vase with carved chrysanthemum motif and elongated neck 天蓝釉刻菊花纹长颈瓶 (From National Museum of China)
	Green		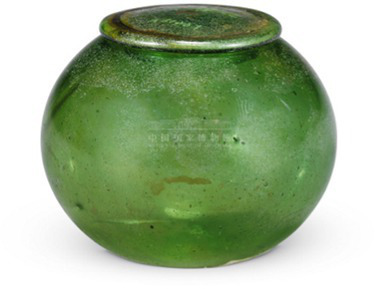 Sui Dynasty Green Glass Lid Jar 绿玻璃盖罐 (From National Museum of China)
	Blue + Green		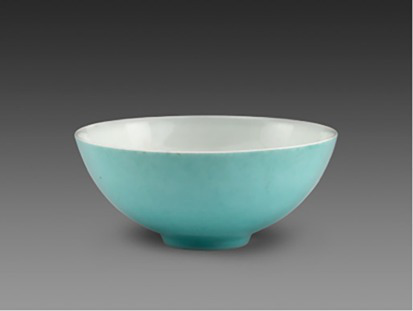 Qing Dynasty Turquoise green-glazed bowl from Jingdezhen kilns 景德镇窑松石绿釉碗 (From Shanghai Museum)
	Pink	Schauss (1979) Annamary (2016)	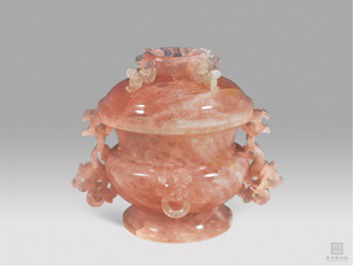 Qing Dynasty Qianlong Rose Quartz Panchi-Eared Covered Censer 乾隆芙蓉石蟠螭耳盖炉 (From Nanjing Museum)
Visual shape	Round (with curves, curves)	Zhou et al. (2021) Geratowski (2020)	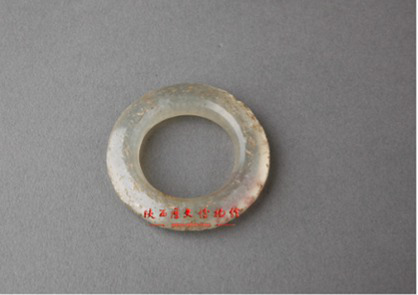 Han Dynasty Jade Ring 玉环 (From Shaanxi History Museum)

Symmetry	Rhodes (2006) Bertamini et al. (2013)	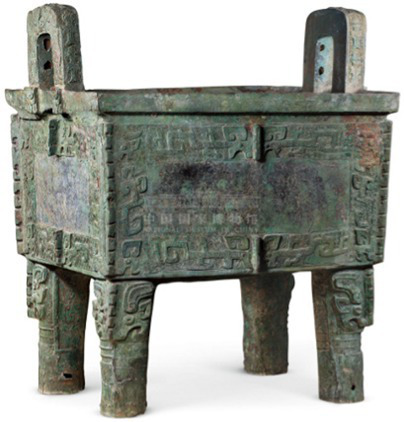 Shang Dynasty Houmuwu Square Cauldron “后母戊”青铜方鼎 (From National Museum of China)

Fractal patterns	Taylor (2006) Voss and Wyatt (1993) Huang and Balsys (2009)	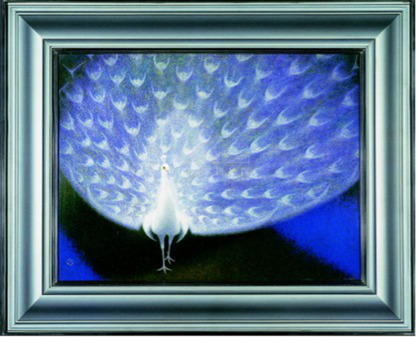 The painting ‘Rhythm’ by Sugiyama Yasushi, gifted by Tanaka Kakuei to Zhou Enlai (From National Museum of China)

Simple/Complex and Innovative contours	Palmer et al. (2013) Salgado-Montejo et al. (2015) Geratowski (2020)	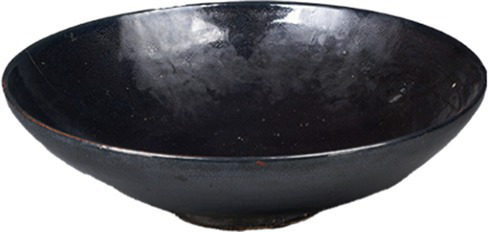 Song Dynasty Black-glazed ‘oil-spot’ bowl 黑釉“油滴”碗 (From Shaanxi History Museum) 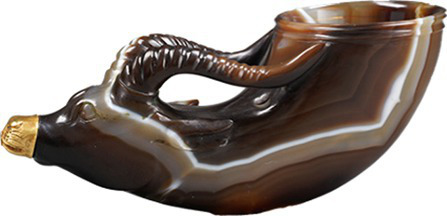 Tang Dynasty Gold-Inlaid Animal-Head Agate Cup 镶金兽首玛瑙杯 (From Shaanxi History Museum)

**Table 5:** List of tactile categories.

Category	Element/variable	References	Collections from museums
Material property	Smooth/rough	Koole et al. (2013) Soranzo et al. (2018)	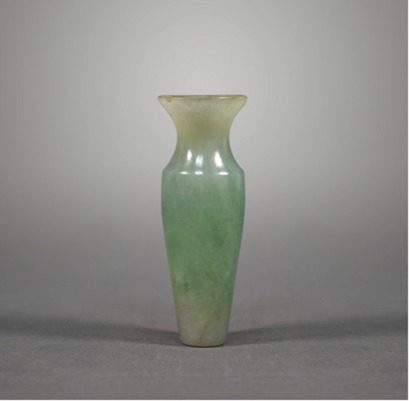 Modern Emerald Flask 翡翠小瓶 (From Shanghai Museum) 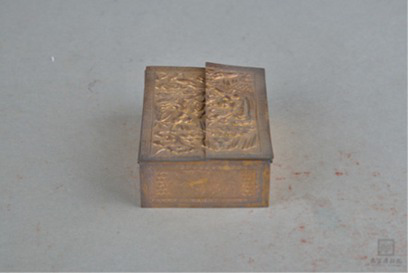 Qing Dynasty Gold-Plated Silver Powder Box with Engraved Flowers 镀金刻花银粉盒 (From Nanjing Museum)
	Soft or wooly	Nimer and Lundahl (2007) Van Horen and Mussweiler (2014) Pasqualotto et al. (2020)	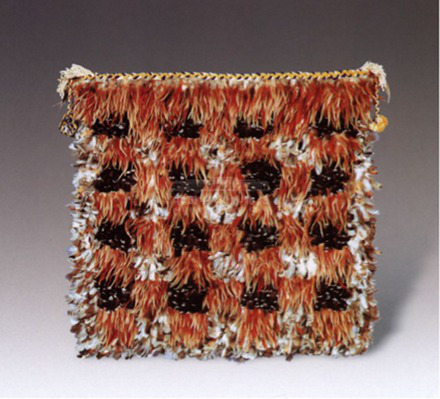 Modern New Zealand feather cloak (From National Museum of China)
	Cold	Shevchuk (2008)	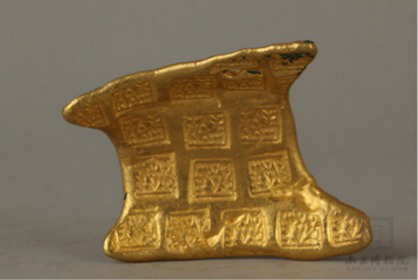 Warring States Period Gold ‘Ying Yuan’ Currency 金郢爰 (From Nanjing Museum)
Geometric property	Rounded	Blazhenkova and Kumar (2018)	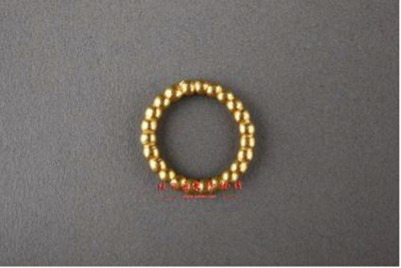 Qing Dynasty Beaded Ring 连珠形指环 (From Shaanxi History Museum)

**Table 6:** List of religious symbols.

Category	Element/variable	References	Collections from museums
Religious symbols	Buddha	Qin and Song (2020) Clobert (2021)	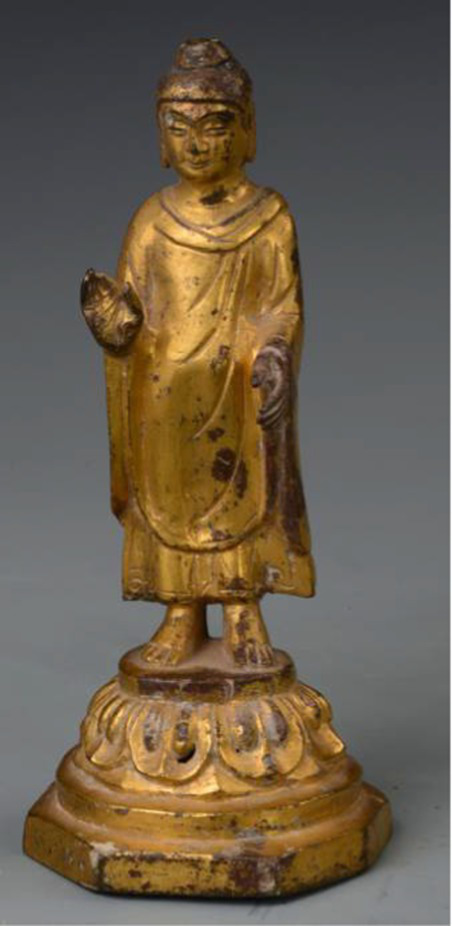 Tang Dynasty Gilded Bronze Buddha Standing Statue 鎏金铜佛立像 (From Gansu Museum)
	Bodhisattvas (Especially Avalokitasvara/willow branches)	Bailey (2009) Kowen (2019)	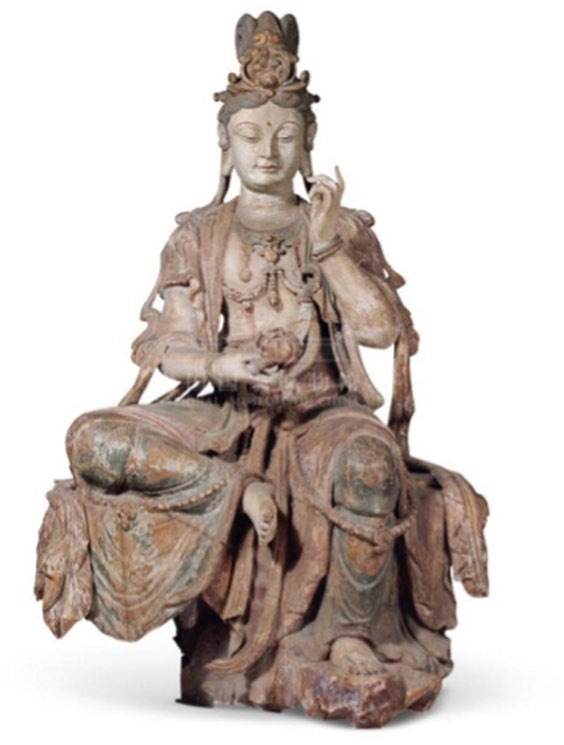 Song Dynasty Polychrome Wood-Carved Seated Guanyin Bodhisattva Statue 彩绘木雕观音菩萨坐像 (From National Museum of China)
	Eight auspicious symbols (conch, Dharmachakra, parasol, victory banner, lotus, vase, pair of golden fish, and endless knot)	Anh and Tin (2021)	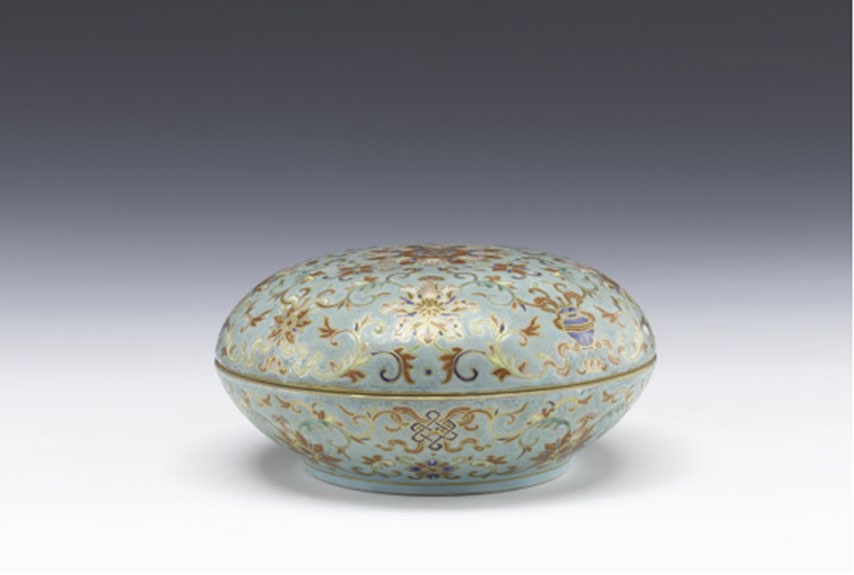 Qing Dynasty Jingdezhen Polychrome and Gilded Porcelain Box with Eight Auspicious Symbols 景德镇窑粉彩描金八吉祥纹盖盒 (From Shanghai Museum)

**Table 7:** List of mythological symbols.

Category	Element/variable	References	Collections from museums
Mythological symbols	Heroes	Bolen (1984) Allison and Goethals (2014, 2016)	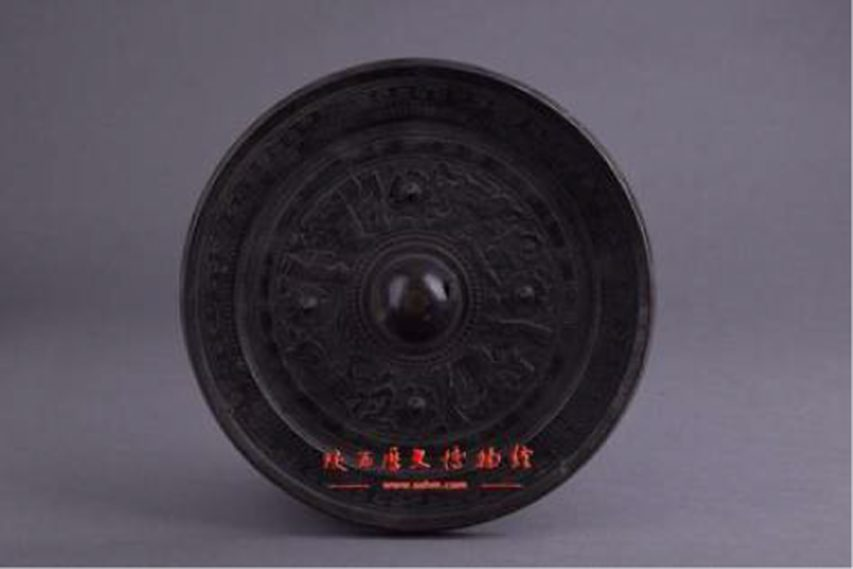 Qing Dynasty Imitation Eastern Han Bronze Mirror with Immortal and Chariot Procession Pattern 仿东汉神仙车马画像纹铜镜 (From Shaanxi History Museum)
	Animals (snakes, auspicious animals, etc.)	Burkert (1996) Jiang (2004) Kim (2020)	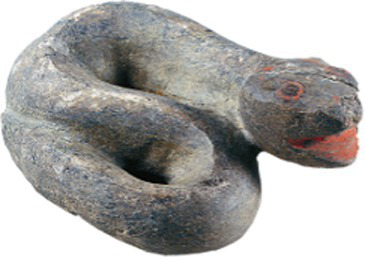 Shang Dynasty Stone Snake (From Jinsha Site Museum)

**Table 8:** List of story tone categories.

Category	Element/variable	Reference	Collections from museums
Story tone and mood	Joy Stories are delightful and interesting (anecdotes)	Xie et al. (2022) Du (2020) Zhang et al. (2017) Wang (2015)	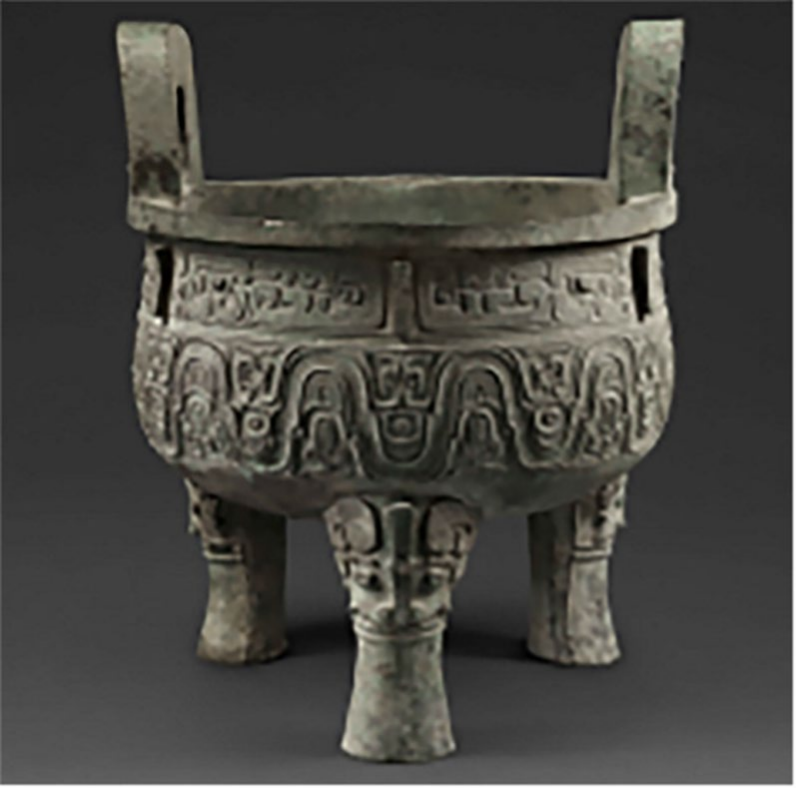 Western Zhou Dynasty Da Ke *ding* 大克鼎 (From Shanghai Museum)
	Anger Stories are outrageous (e.g., items are damaged and stolen)		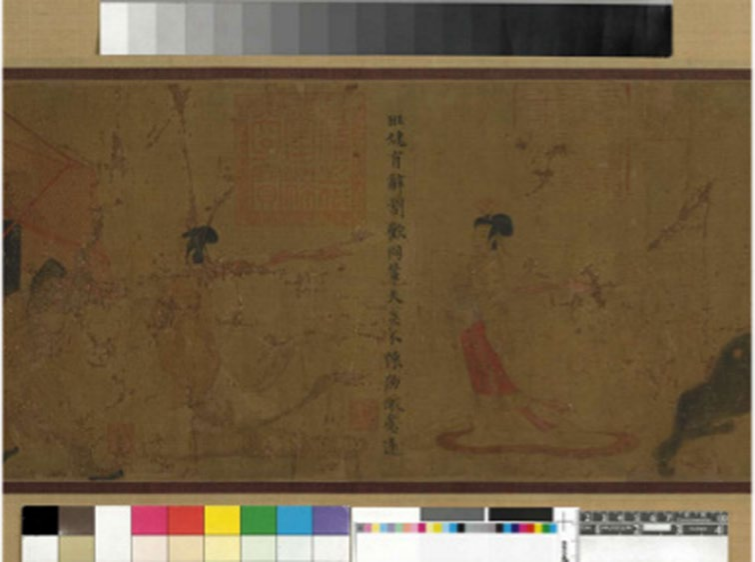 Tang Dynasty *Admonitions Scroll* 女史箴图 (From the British Museum)

**Table 9:** Story topic and connotation categories.

Category	Metavariables	Reference materials	Relevant collections of major museums
Story topic and connotation	Love (love, friendship, affection, etc.)	Sheih (2014) Jiang et al. (2022)	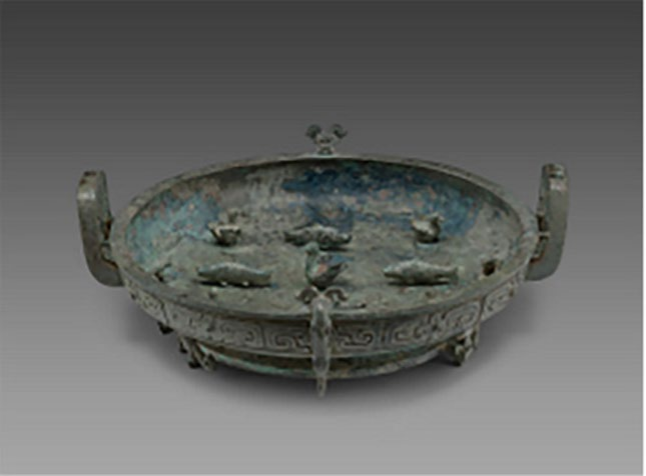 Spring and Autumn period Pan (water vessel) of Zi Zhong Jiang 子仲姜盘 (From Shanghai Museum)
	War	Innes and Sharp (2021) Wang and Wu (2022)	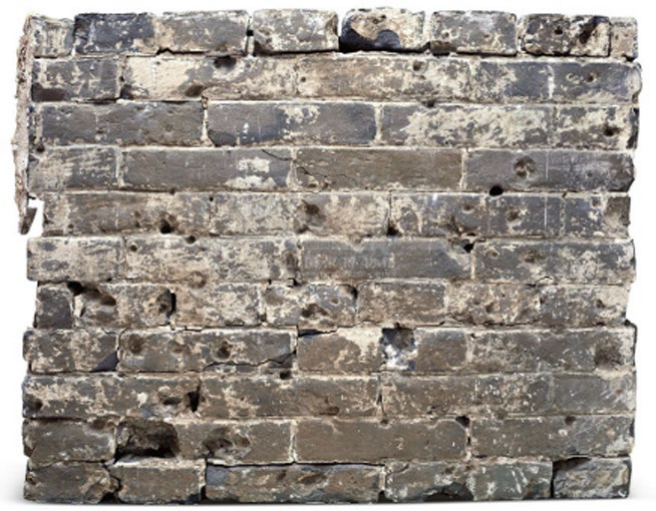 Bullet-Scarred Wall from the Battle of Taierzhuang (From National Museum of China)
	Disease	Sontag (2011) Koster and Baumann (2005)	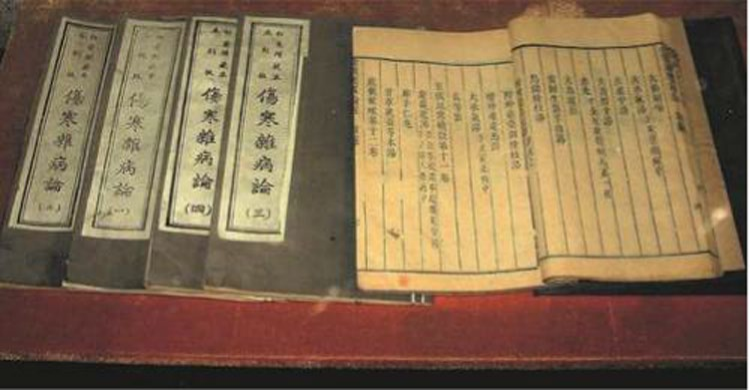 Republic of China The Baiyun Pavilion Edition of ‘Treatise on Cold Damage and Miscellaneous Diseases 白云阁本《伤寒杂病论》 (From Zhangzhongjing Museum)
	Childhood memory	Hepper et al. (2012) Zhou et al. (2008)	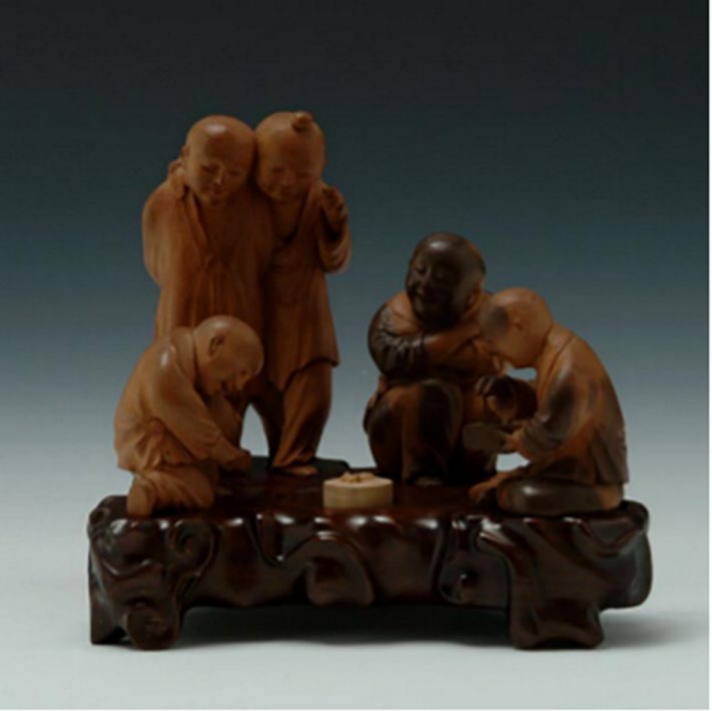 Modern Boxwood carving of children playing with a cricket 朱子常黄杨木雕群童蟠戏 (From Shanghai Museum)
	Collective memory	Yim (2022)	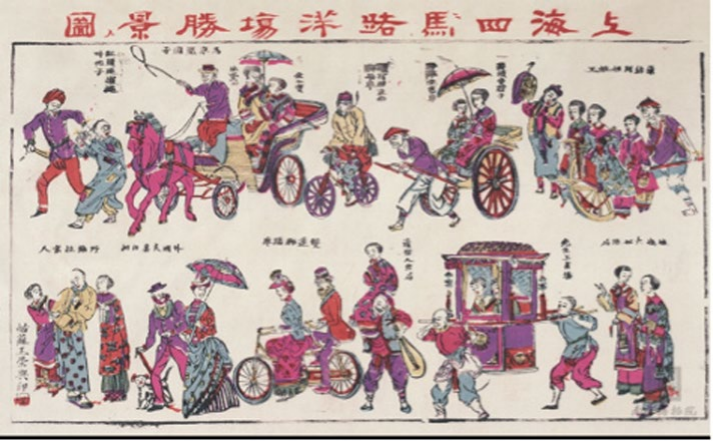 Qing Dynasty Suzhou Taohuawu New Year’s Picture: ‘Scenic View of the Fourth Road in Shanghai’ 苏州桃花坞年画《上海四马路洋场胜景图》 (From Nanjing Museum)

Based on the aforementioned elements of museum healing objects, the authors selected seven items from the Shanghai University Museum for use in the workshop (Table 10). These items included the student card of Tian Shenhua, an archive major at the College of Liberal Arts of Shanghai University; the exercise book from Aurora University; the 1939 annual grade report (transcript) for students of Gezhi Middle School; the 1952 report on the living conditions of the kindergarten at the privately-run Guangming Primary School; the modern painting “Tired Journey” by Feng Zikai; replica rap figurines from the Chengdu Museum; and white porcelain from the Xing Kiln (Figure 1). These seven collections were all mentioned in the focus group interviews with the visitors. Based on appraisal theories of emotion, the authors summarized the psychological responses of visitors to the collections as revealed in the interview content. Table 11 shows the visitors’ various emotional characteristics related to each collection. First, the authors anonymized all transcripts by replacing visitor names with unique codes (i.e., 20231124C) to ensure traceability and confidentiality. Second, the authors extracted all visitor statements related to the seven objects from the interview texts and identified explicitly expressed or implicitly conveyed emotions and attitudes in these statements. Each statement was assigned a preliminary “emotion label.” For example, regarding “Tired Journey,” the visitor’s comment “the person in the painting looks very lonely” was coded as “sympathy,” while “his state makes me feel relaxed” was coded as “comfort and ease.” Third, all emotion labels from the second step were consolidated and categorized to form the final “emotional characteristics” dimension, as presented in Table 11. Simultaneously, the number of visitor statements expressing the same emotional characteristic was counted, resulting in the data for the “frequency of visitor mentions” column in the table (i.e., “Tired Journey” was mentioned 10 times in total, with 2 of these mentions expressing “heartache and sympathy”).

**Table 10:** Collection selection criteria for the museotherapy workshops at Shanghai University Museum.

Objects	Description	Photo	Element	Category
The student card of Tian Shenhua, an archive major at the College of Liberal Arts of Shanghai University	The 1985 student ID card for an archival studies major records the basic family and employment information of Tian Shenhua, a student at the time. Tian Shenhua’s age and life experience align with those of the target audience’s grandparents, giving the artifact strong attributes of collective memory. It thus meets the selection criteria for objects belonging to the “collective memory” category within the psychological dimension and can evoke resonance among visitors with their grandparents’ experiences.	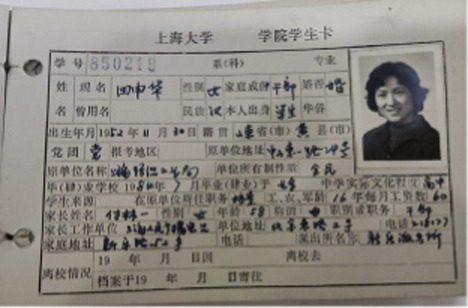	Collective memory	Psychological dimension
The exercise book from Aurora University	As a historical educational artifact, this exercise book contains a student’s chemistry class notes. Both its content and form closely align with the student identity of the participants; effectively evoking memories related to school life. It thus fulfills the criteria of the psychological dimensions of “childhood memories” and “collective memory.”	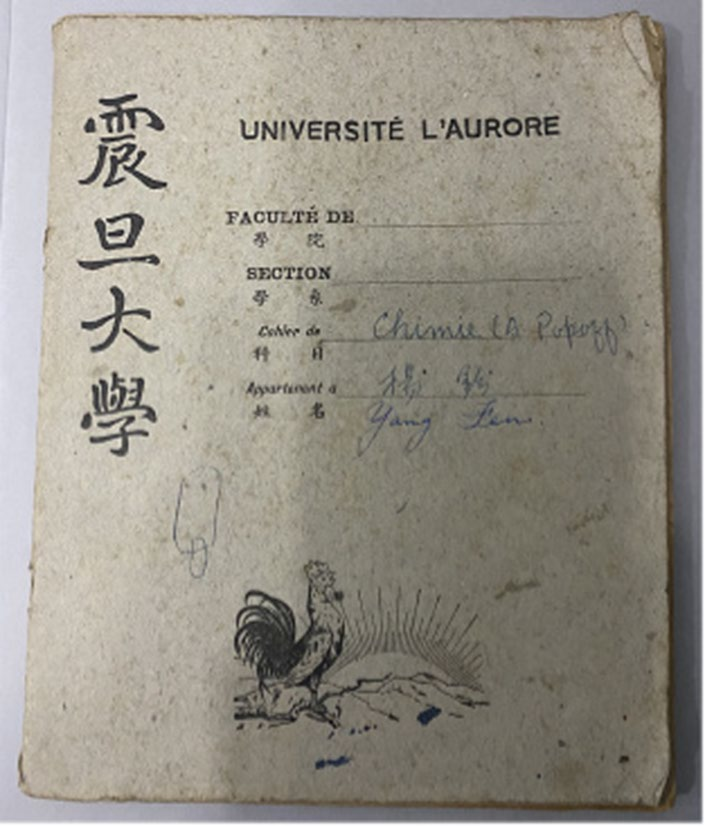	Childhood memory and Collective memory	Psychological dimension
The 1939 annual grade report (transcript) for students of Gezhi Middle School	Similar to the previous artifact, as a quintessential object from one’s student years, the transcript naturally evokes school-related memories among viewers. When contextualized within its historical background, it further reinforces collective memory, thereby meeting the selection criteria within the psychological dimension.	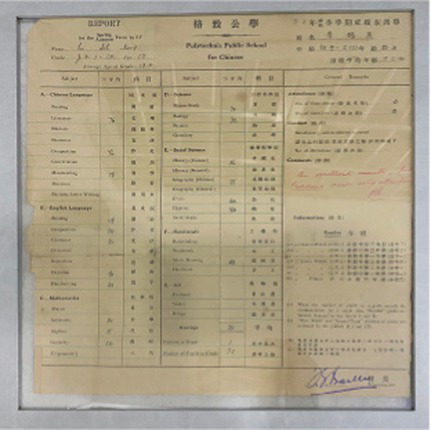	Childhood memory and collective memory	Psychological dimension
The 1952 report on the living conditions of the kindergarten at the privately-run Guangming Primary School	This report reflects early childhood education and daily life in 1950s China, effectively evoking viewers’ related childhood memories and collective memory. It thus aligns with the relevant healing elements within the psychological dimension.	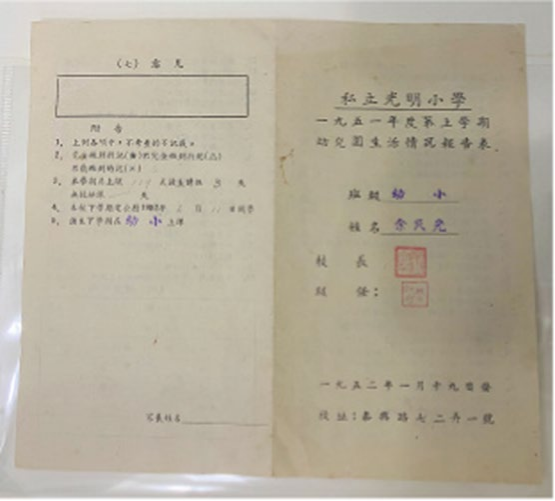	Childhood memory and collective memory	Psychological dimension
The modern painting ‘Tired Journey’ by Feng Zikai	The painting uses the “journey” as a metaphor for life’s state, resonating with viewers on topics such as life values, self-reflection, and love for life and oneself. Its themes touch upon love and collective memory, aligning with the emotional guidance objectives for artifacts in the “Love” and “Collective Memory” categories within the psychological dimension.	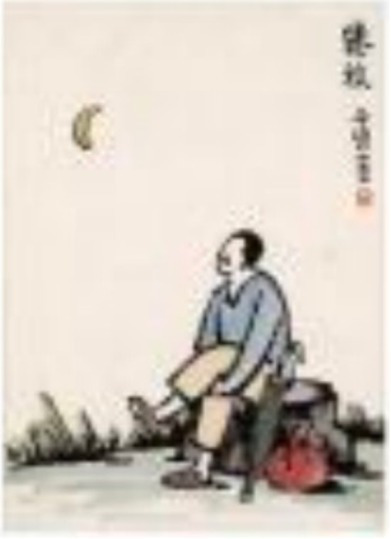	Love and collective memory	Psychological dimension
Replica rap figurines from the Chengdu Museum	With its rugged form, coarse texture, and complex yet innovative contours, the piece aligns with the healing elements of “roughness, coldness, and complex, novel contours” within the physical dimension.	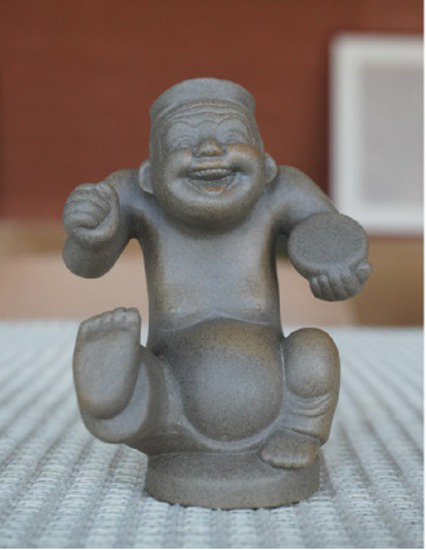	Rough and cold and complex and innovative contours	Physical dimension
White porcelain from the Xing Kiln	With its smooth surface, cool tactile sensation, and simple yet innovative form, this piece aligns with the healing elements of “smoothness, coldness, and complex, novel contours” within the physical dimension.	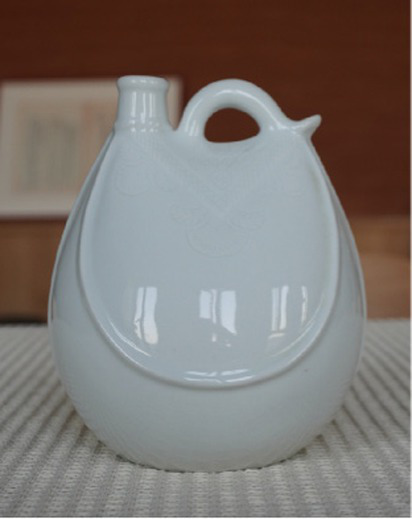	Smooth and cold and complex and innovative contours	Physical dimension

**Table 11:** Visitors’ emotional characteristics related to each collection.

Collections/related creations	Healing objects elements	Visitor mentions	Visitor emotional characteristics
“Tired Journey”	Psychological element: the theme of love and collective memory	10	Some visitors felt distressed and sympathized with the loneliness of the people in the painting; some were curious about what kind of path Feng Zikai would embark on if he did not paint. Some sympathized with the nostalgia or worries and anxiety of the people in the painting, while some believed that the people in the painting were in a relaxed state. It made some people feel comfortable and relaxed; the visitors even reconciled with themselves after appreciating the painting.
Secondary creation of “Tired Journey”		12	Six of the visitors stated that they wanted to help the person in the painting, so they drew the sun, the shade of a tree, companions, family members, etc., for the person in the painting, and hoped that he would no longer be alone and could be happy. The stories of the collection and the painter aroused the visitors’ “love” for their hometowns and lives and for themselves.
1939 annual grade report (transcript) for students of Gezhi Middle School	Psychological elements: childhood memory and the collective memory	20	The visitors expressed love, sympathy, and regret for the students during the war, while some were filled with hope for them. Reflecting on their own future student lives, they envied the richness of academic life in the past. However, some visitors noted that the transcripts also stirred feelings of anxiety. Through the transcripts and the stories behind them, visitors recognized the challenges of studying in earlier times and expressed gratitude for their current circumstances.
Aurora University exercise book	Psychological elements: childhood memory and the collective memory	14	The Aurora University exercise book evoked emotions among the visitors that were similar to those associated with the transcripts.
Tian Shenhua’s student card	Psychological elements: collective memory	9	The owner of Tian Shenhua’s student card is married and is somewhat older. From Tian Shenhua’s life trajectory and work experience, the visitors expressed admiration and appreciation for her, viewing her as a role model. Some visitors even had relatives who worked at the same organization as Tian Shenhua, yet their paths were vastly different, prompting reflections and curiosity about what kind of university experience Tian Shenhua had while attending college in the 1980s.
Rap figurines	Physical elements: rough to the touch, cold, complex and novel contours	6	The visitors expressed sympathy for the plight of the ancient rap comedians, but at the same time, they were touched by the smile of the rap figurine, feeling a sense of warmth and happiness. This was the most direct piece among all the selected collections that could evoke a feeling of ‘happiness’ in the visitors. As one visitor noted, “I held this pottery figurine to feel for a while and then I realized that he belonged to the Chengdu Museum. Because I am from Chengdu, I immediately felt that he was very kind.” Visitors can touch this pottery piece, making it easier to establish an emotional connection with the collection.
Xing Kiln white porcelain	Physical elements: smooth to the touch, cold with novel contours	4	The visitors expressed their sense of novelty toward the white porcelain, and one even noted, “When I saw this piece of white porcelain, I felt that it was very crystal clear. It was heavy in the hand and felt very comfortable.”

For museum visitors, heritage objects can carry symbolic meaning for individuals if feelings are projected onto them. The object can act as a repository or container for projections of different states of mind (Lanceley et al., 2012). When the emotional evaluation process of a visitor is connected with the object, the individual emotion dominated by the object begins to change. The inter- and intraindividual variation in the appraisal process and the variables contributing to it will result in different emotion-related activities. Each variable contributes to the appraisal underlying the emotional response and its regulation by coping. As such, these appraisals contribute to inter- and intraindividual differences in emotional response (Lazarus, 2021).

In summary, to effectively engage the emotional resonance of young visitors through museum objects, this paper suggests that it is essential to first clarify the demographics and profiles of the target visitors. Subsequently, within the framework of healing objects, we should seek out items that resonate with these visitors. The collections can facilitate a deeper understanding of their meanings and significance. At the same time, visitors will unconsciously bring in objects or shapes related to “people” (such as personal items such as Tian Shenhua’s student card or humanoid objects such as rap figurines), generating a richer emotional experience and personal connection. Notably, directly touching objects amplifies visitors’ sensory experiences. The emotions evoked by the senses—such as novelty, comfort, and happiness—are often more straightforward and intense than other emotions.

### 3.3 Narrative-based dialogue framework in the interviews

In recent decades, psychology has increasingly focused on the uniqueness of the individual, showing a keen interest in life stories and their myriad possibilities. Furthermore, the diverse ways in which individuals interpret their life experiences and derive meaning from them have garnered significant attention. In this context of the “narrative turn” in psychology (Vassilieva, 2016), narrative-based dialogue framework has emerged as a vital approach.

In narrative-based dialogue, psychotherapists employ various distinctive methods to enhance the effectiveness of their conversations, and elements of these techniques can be observed in the interactions between the psychological counselor and visitors during the workshop (Table 12). Consequently, it is crucial to structure a clear pathway for visitors to achieve a meaningful emotional experience efficiently.

**Table 12:** Use of narrative-based dialogue framework in interviews.

Therapy skills	Specific use	Effect analysis
Externalizing conversations	“The problem becomes the problem, not the person (White, 2024).” This is the core idea of the practice of externalizing conversations in narrative therapy. When the problem becomes an entity that is separate from the person, and when people are not tied to restricting “truths” about their identity and negative “certainties” about their lives, new options for taking action to address the predicaments of their lives become available. Rather, if the person is the problem there is very little that can be done outside of taking action that is self-destructive (White, 2024). In the externalizing conversations of psychological counselors, metaphors often appear as a way to help people redefine themselves and their problems.	In the workshop, the psychological counselor will guide the visitor to imagine the specific event of anxiety as an object. “You can imagine this thing that usually brings anxiety to you as an object. What kind of object is it? What shape? Color? Texture? Can you imagine?” Participants may come up with a variety of responses, such as a mountain, a black hole, mud stuck to their shoes, countless rays directed at themselves, and so on. This process guides the visitors to first externalize their worries, detach themselves from them, and then engage in externalized dialogue. After the visitors have finished answering, the psychological counselor explained the reasons for this approach: “The reason everyone envisions this image is that the anxiety we all feel stems from a situation that seems unsolvable, making it difficult to view it from an outsider’s perspective. However, by representing it as a concrete image, we often gain greater clarity. Once we recognize its impact, we can approach the situation from a more rational standpoint. For instance, as one just shared, his troubles are akin to a mountain; at times, the mountain feels smaller, and at other times, it looms larger. We can also try to internalize this mountain, transforming it into something that we can engage with—something we can work to resolve.” At this point, the two parties in the dialogue have completed a relatively straightforward process of externalization triggered by metaphor, allowing the visitor to consciously learn to view their problems from the different perspective of an “outsider.”
Outsider-witness	In narrative-based dialogue, there is a role called “outsider-witness” (White, 2024), outsider witnesses engage one another in conversations about the expressions of the telling they were drawn to, about the images that these expressions evoked, about the personal experiences that resonated with these expressions, and about their sense of how their lives have been touched by the expressions. In these outsider witness retellings, what people give value to in their acts of living is represented in ways that are powerfully resonant and highly acknowledging. When a person poses a question but does not receive an answer, a significant reason often lies in their own limited way of thinking. Accepting feedback from others about the question helps them examine their stance when facing the issue. By reflecting on, analyzing, and distinguishing their insights from those of others, the questioner gradually clarifies their position. Everyone in a focus group serves as an “outsider-witness” to the others. During the conversation, the therapist often emphasizes the diversity of answers and the impact of that diversity on individuals, which is also a common supportive technique used by counselors.	In the workshop, a common question posed by the psychological counselor is, ‘What do others think about this?’ or ‘What different answers do others have?’ They might also directly ask the topic initiator, ‘Has what others have shared inspired you?’ This approach encourages a diversity of responses. For instance, when the counselor asks about ways to cope with anxiety, some participants mention exercising, others speak of self-reflection, and some suggest taking action to find solutions. On the surface, everyone is sharing their coping methods, but this also leads to insights and reflections brought about by different perspectives. Such focus group discussions help participants realize that they share similar concerns, thereby alleviating feelings of loneliness.
Reauthoring conversations	“Great storytelling … is about compelling plights that … must be set forth with sufficient subjectivity to allow them to be rewritten by the reader, rewritten to allow play for the reader’s imagination” (Bruner, 1986) Everyone’s life is an unfinished story, which also contains nodes and clues. However, due to the biases and preferences of individual memories, clients are unable to objectively recognize every node and clue. At this point, the counselor needs to help them become aware of those events or experiences that have been overlooked yet hold extraordinary significance, enabling them to rewrite their story. These events and experiences can be considered “unique outcomes” or “exceptions (White, 2024).”	In the workshop, the psychological counselor will ask the visitor to write down their recent worries, the things they want to forget, the recent happy things, and the recent changes they want to make. This is the process of allowing the visitor to reorganize the “story fragments,” suggesting the “exceptions” that have been neglected by themselves. The act of writing it down also makes the memory more objective. One of the visitors said after writing about her recent happy events: “I feel as if there are more happy things than I imagined. In addition, after I emphasized it (the happy things), I found that I was doing well.” At this time, the visitor has already discovered the “exceptions,” and they are a new entry point for her rewriting this fragment of the story.
Landscape of consciousness	Bruner, borrowing significantly from the literary theorists Greimas et al. (1976), proposed that stories are principally composed of two landscapes—a “landscape of action” and a “landscape of consciousness.” The landscape of action is the “material” of the story and is composed of the sequence of events that make up the plot (*sjuzet*) and the underlying theme (*fabula*). The landscape of consciousness is composed of “what those involved in the action know, think, or feel, or do not know, think or feel” (Bruner, 1986, p. 14). In a literary work, readers infer characters’ intentions and purposes through the plot, thereby deducing their personality traits and entering the blueprint of consciousness. In this process, not only the thoughts of the characters and the author are reflected, but it also mirrors the readers’ own thoughts and consciousness.	In the workshop, we regarded the museum objects as different literary works. When ‘reading’ these objects, each visitor brought their own thoughts and consciousness into the experience. The details of the stories were shaped by the visitors’ memories and life experiences, creating a sense of resonance. In this way, by using the narratives of the objects as an entry point, visitors established a ‘story consciousness.’ Consequently, it became more coherent to use these objects to guide the visitors’ personal narratives, further reinforcing the connection between their stories and those of the objects. This is why objects serve as some of the most significant emotional triggers within the workshop.

## 4 The “W” emotion curve: video analysis results

In emotion studies, in addition to sampling emotional sentences, action expressions can be sampled to determine the instantaneous emotion of an individual. Ekman noted that motion records (film or video) are preferable to still photographs (Ekman et al., 1972). Moreover, based on the emotional expression of facial expressions in daily life, he proposed six basic expression examples, including happiness, sadness, surprise, fear, anger, and distress (Ekman and Friesen, 1978). He proposed the facial affect scoring technique (FAST) which involves scoring each observable movement in each of three areas of the face: (l) the brows/forehead area; (2) the eyes/lids; and (3) the lower face, including the cheeks, nose, mouth, and chin. In terms of the scoring category, FAST employs photographic examples to define each of the movements within each area of the face, which, theoretically, distinguishes among six emotions: happiness, sadness, surprise, fear, anger, and distress (Ekman et al., 1972).

Therefore, we can utilize visitors’ facial expressions to further refine the emotional classification derived from text analysis. Simultaneously, we can analyze and interpret the emotions expressed through their non-verbal cues (Table 13). Given that the overall mood of the workshop remained predominantly neutral, without any extreme emotions such as anger or fear, we can also consider body movements to enhance our understanding of the visitors’ emotional states.

**Table 13:** Description of emotional intensity levels.

Expression analysis	Emotional intensity	Video screenshot
Eyebrows raised, eyes squinted and partially closed, nose wrinkled, and corners of the mouth turned upward to reveal teeth.	Strongly positive	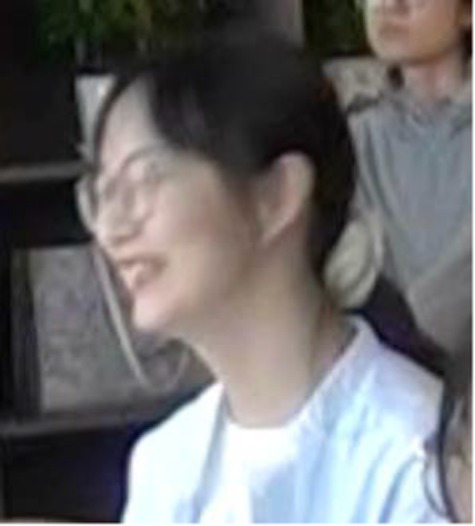
Eyebrows raised, eyes slightly narrowed, nose slightly wrinkled, and corners of the mouth upturned.	Moderately positive	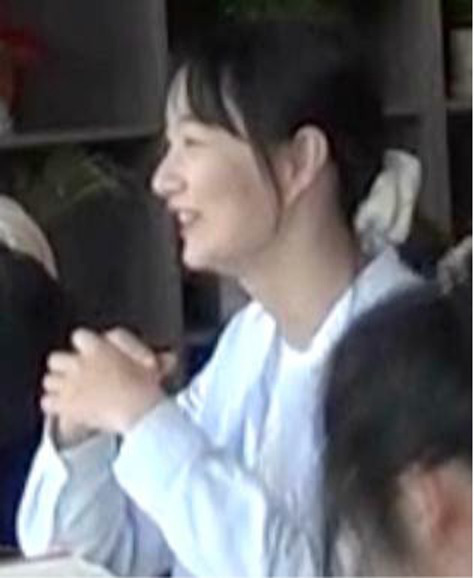
Eyebrows remained still, eyes gazed straight ahead, and the corners of the mouth showed no movement.	Neutral	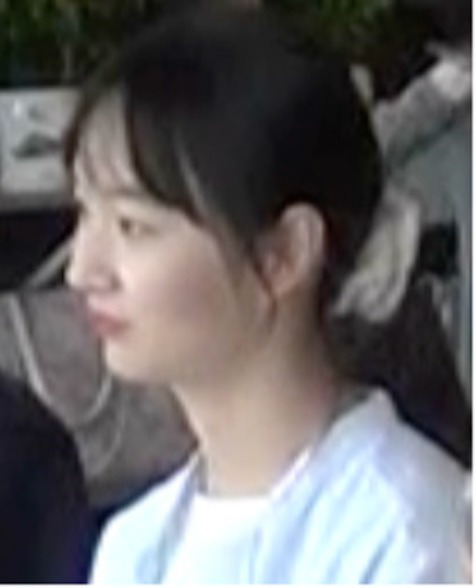
Eyebrows slightly furrowed, eyes gazing straight ahead, and the corners of the mouth turned slightly downward.	Moderately negative	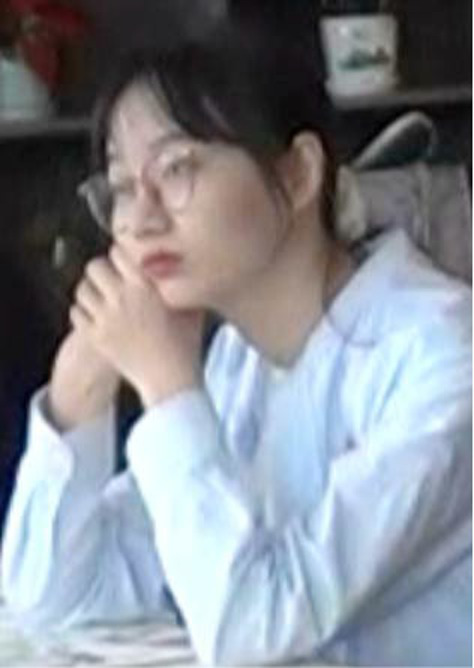

The following is an example of text with a known positive emotional tendency:

Upon seeing this exercise book, I was instantly transported back to my high school days, and my inner student reemerged. Just as I used to doodle and scribble in the margins of my textbooks, I found myself drawing beside the illustrations once again. Oh, look! I can see the owner wrote “cannot you” next to the doodle.

According to appraisal theories of emotion, the visitor experienced happiness upon encountering the emotional trigger—the exercise book. After reflecting on it, she associated it with her high school days, which evoked pleasant memories of doodling in the margins. When she remarked, “It feels like being back in high school,” this visitor (No. 231107C) smiled broadly, her eyes crinkling with joy, and by the end, she was laughing out loud, covering her mouth with her hands. This indicates that the refined emotion correlating with the positive emotional text should be happiness. As illustrated in Tables 14, 15, we captured snippets of dialogue, facial expressions, and physical behaviors from both a boy and a girl during their participation in the workshop to analyze the emotions expressed through both verbal and non-verbal communication.

**Table 14:** Table of emotions generated from activities by visitor 231013D (male, sophomore).

Activity process	Part of the interview text (emotional tendency)	Expressions and body movements emotions expressed	Video screenshot
Icebreaker game		Laughed several times during the game	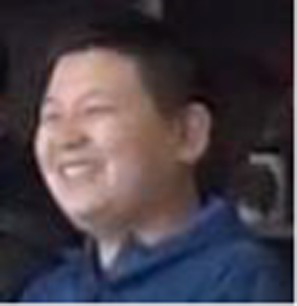
Observe and touch the collections up close		Serious look, thoughtful, sometimes smiling	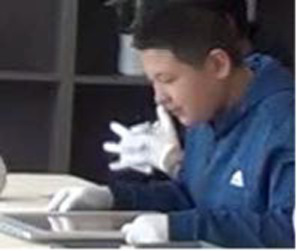
The host tells the story of the collection		Listen carefully	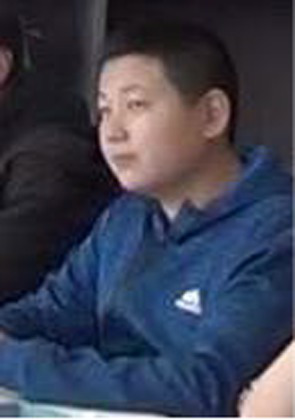
Interview with a psychological counselor		Mainly listens; will be amused when other participants bring up some topics	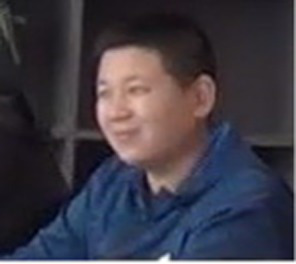
Write down your recent changes/troubles		Thoughtful	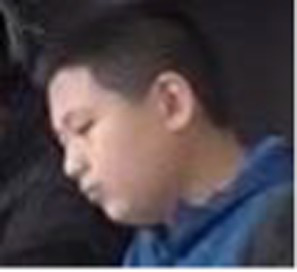
Talk about your troubles	“I hope to be able to dedicate more attention to a single task. When faced with multiple responsibilities, I find it hard to concentrate on one thing, so I aspire to be more focused. I believe this is achievable; for instance, by creating a conducive environment and keeping my phone out of reach.” (positive)	Sometimes frowned slightly	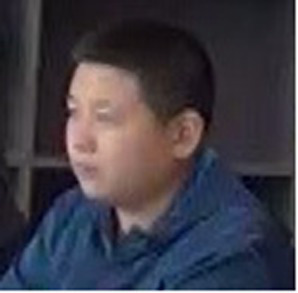
Listen to other’s views on their troubles		Sometimes nods in agreement	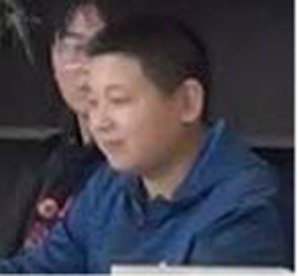
Art creation		Serious creation	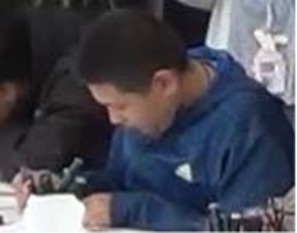
Communication after the art creation is over	“Today’s biggest gain was expressing my worries. After sharing, I even felt a sense of enlightenment; I had previously never really paid much attention to my own feelings.” (positive)	Smiling and nodding frequently	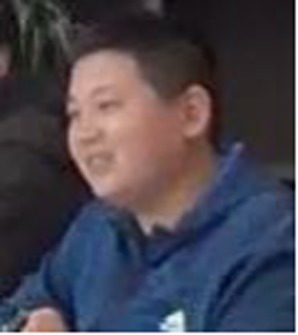
Receive a small gift after the workshop		Happy and grateful	(Camera did not shoot)

**Table 15:** Table of emotions generated from activities by visitor 231013C (female, sophomore).

Activity process	Part of the interview text (emotional tendency)	Expressions and body movements emotions expressed	Video screenshot
Icebreaker game		Laughed several times during the game	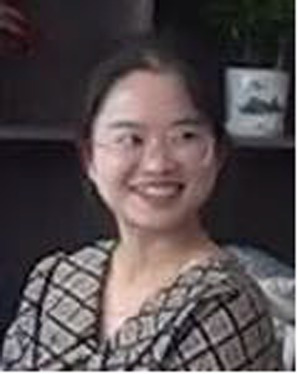
Observe and touch the collections up close		Sometimes, with her hand on her cheek and a curious expression	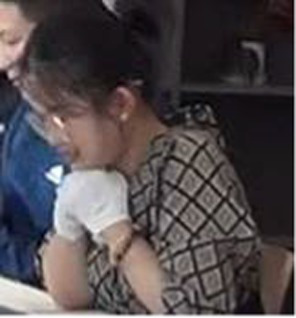
The host tells the story of the collection		She rested her cheeks on her hands and listened attentively	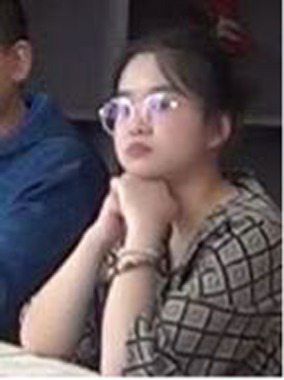
Interview with a psychological counselor	“I would like to ask Feng Zikai, if he was not engaged in fine arts, what other choices would he have?” (positive)	She used gestures to assist her speech, was eager to express herself, and started to smile toward the end	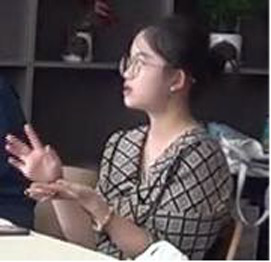 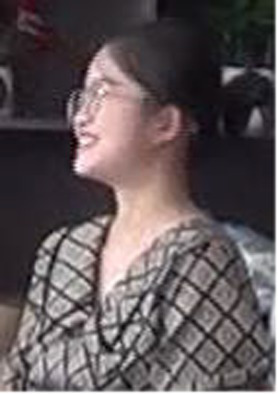
Write down your recent changes/troubles		Concentrated on writing without thinking	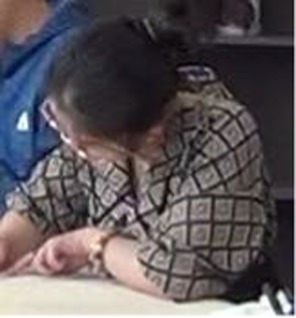
Listen to other’s views on their troubles	“If you are unhappy, you can make more friends. If they are friends with similar interests, they can accompany you to do many things. Sometimes doing things together will relieve you a lot.” (positive)	Engaged in communicating with the psychological counselor, nodding while talking	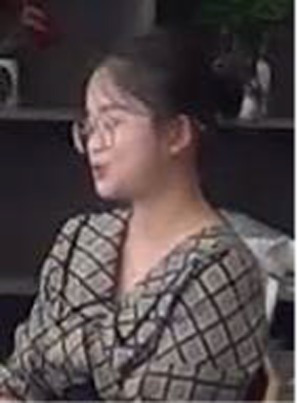
When the psychological counselor puts forwards her views and summarizes		Nodding frequently and smiling in agreement	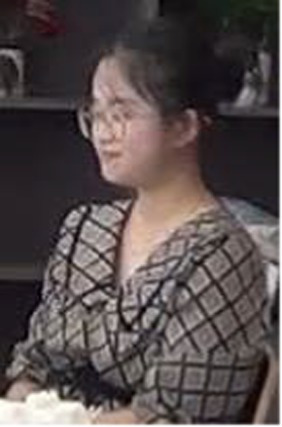
Art creation		Serious creation	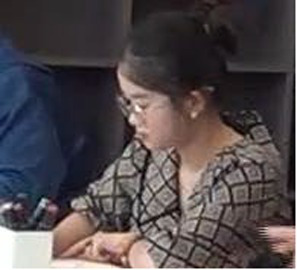
Communication after the art creation is over	“I think it is a special experience to see museum objects up close. Before, I used to see the exhibits in the museum through the showcases, but now they can be placed in front of me to observe and touch very carefully, which is relatively rare.” (positive)	When she was about to finish speaking, she nodded slightly to indicate agreement	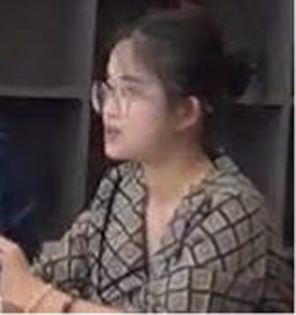
Receive a small gift after the workshop		Happy and grateful	(Camera did not shoot)

**Figure 3:**
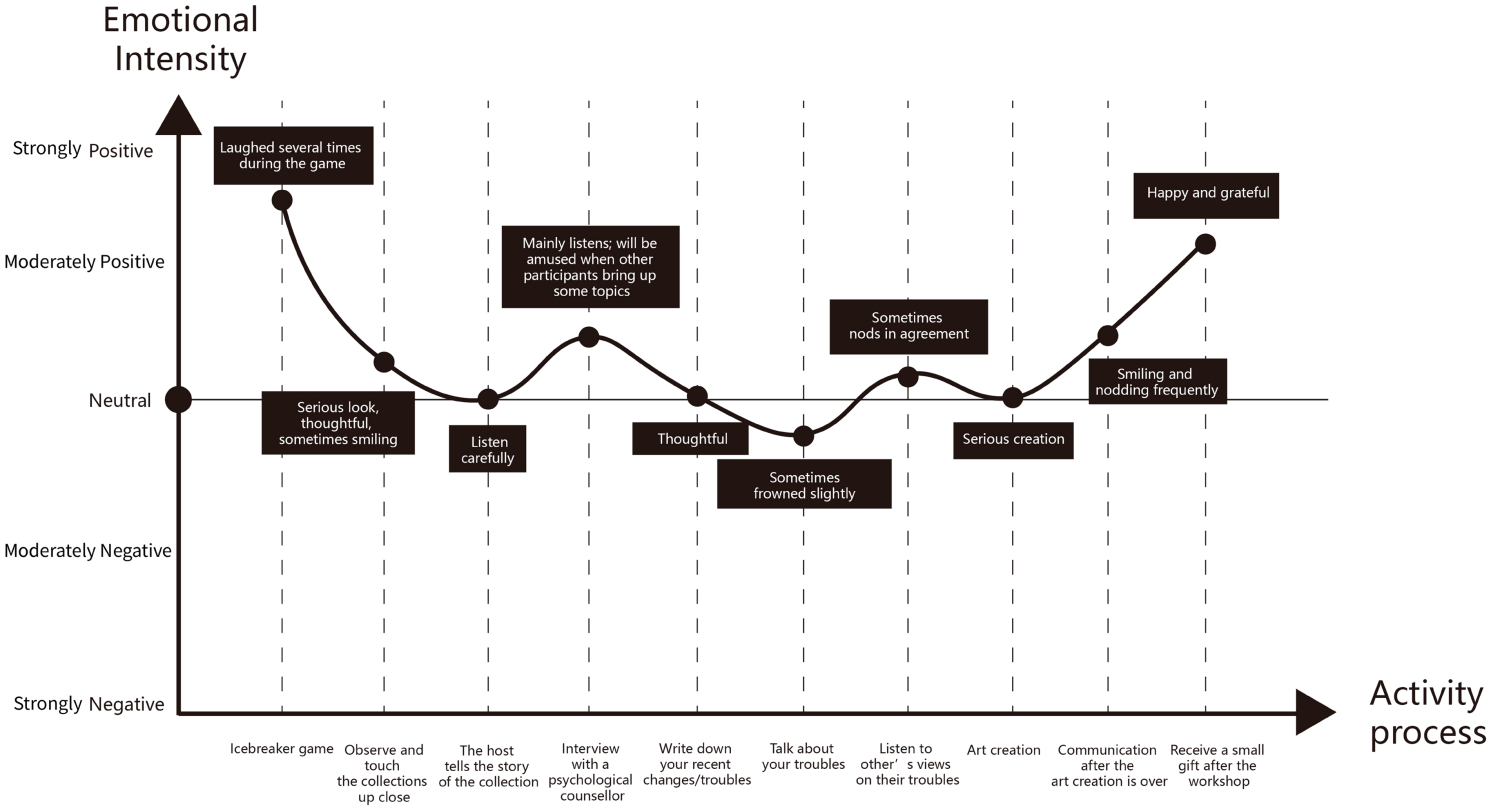
The emotion curve drawn by visitor 20231013D based on the workshop video.

**Figure 4:**
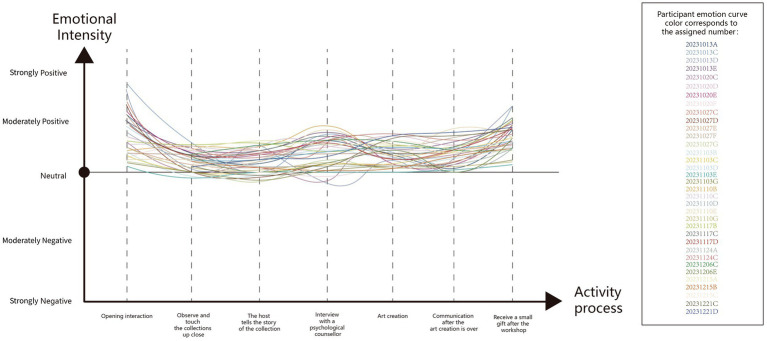
Emotion curve of 36 participants.

By examining facial expressions and body movements, we can more accurately determine visitors’ emotional changes at each stage of the workshop. Based on our observations from the video, we created an emotion curve to systematically analyze the overall emotional trend of each individual (Figure 3). Subsequently, we selected the complete video recordings of the 36 participants^5^ and generated a set of emotion curves (Figure 4) to offer a more comprehensive understanding of the overall emotional experience throughout the workshop.

As illustrated in the emotion curve, the overall emotional tone of the visitors ranged from neutral to positive, with most visitors’ emotional responses remaining fairly consistent; the overall score was “W” (curve shape). From the perspective of the workshop process, in addition to the triggers previously analyzed, the visitors’ emotions were also influenced by their personal preferences for the attributes of the workshop. For instance, some visitors were more reserved and preferred interactions that did not require much communication, while others desired more opportunities for dialogue. Furthermore, as this was a group activity, the visitors’ emotions were also shaped by the feelings of their peers. If no one in a group was good at speaking, the visitors’ emotional responses tended to be relatively subdued.

## 5 Conclusion

This study initially utilized the interview texts extracted from the videos as primary data and employed the SPSSAU to analyze the emotional tendencies of the texts. Subsequently, based on appraisal theories of emotion, the study identified the emotion triggers of positive emotional tendencies within the texts, revealing that the objects and their connotations during the workshop were the primary factors in evoking positive emotions in the visitors. Furthermore, the research further validated the effectiveness of the healing objects elements and indicated that, at the psychological level, collections that are closely related to “people” and highly aligned with personal experiences are more likely to serve as significant emotion triggers for young visitors.

Moreover, this paper provides a detailed analysis of the narrative-based dialogue framework employed in interviews with the psychological counselor. By utilizing externalizing conversations, diversifying responses, and re-authoring conversations, alongside the narrative power of objects, this museotherapy effectively facilitates the expression and release of visitors’ emotions, exploring a positive and constructive pathway for emotional wellbeing. Finally, we utilized the videos recorded during the workshop to observe the expressions and body movements of all participants. By integrating these observations with the interview texts, we conducted a detailed analysis of the visitors’ non-verbal emotional expressions, which led to the creation of the “W” emotion curve. This approach enabled us to gain a deeper understanding of their emotional changes throughout the workshop, revealing that the overall emotional variation among the visitors was minimal, with the majority exhibiting neutral to positive emotions.

In summary, this study explores the potential mechanisms underlying the young participants’ emotional experiences in museotherapy workshops. Through their interactions with museum objects, young visitors not only acquire rich emotional experiences but also actively participate in various activities, such as art creation and interviews. This engagement fosters self-healing and emotional regulation. Although this study has yielded some findings, it still possesses certain limitations. Regarding the research sample, constraints in time and resources led to the selection of only a limited number of participants and cases for analysis, which may affect the generalizability of the research results to some extent. Thus, future studies delving into long-term efficacy and sustained impact of museotherapy activities merit attention. It is hoped that future research can expand the scope of study and employ methods such as biofeedback techniques ^[^^6^^]^ to assess the effects more accurately. Concurrently, it is anticipated that future studies will further explore the influence of different types of cultural heritage and various museum settings on visitors’ emotional experiences, as well as how to enhance the therapeutic outcomes of museums through innovative healing activities. This could involve integrating modern technological means—such as virtual reality and augmented reality—with cultural heritage to create more immersive, multi-sensory museotherapy experiences; or combining museotherapy with other social service programs, such as well-being education and community services, to form a more diversified service spectrum.

## Data Availability

The raw data supporting the conclusions of this article will be made available by the authors, without undue reservation.

## Footnotes

1 SPSSAU is a web-based online data analysis platform. The URL for the SPSSAU online platform is as follows: https://spssau.com/.

2 The URL for the SPSSAU online platform is as follows: https://spssau.com/.

3 According to the platform’s usage instructions, the text sentiment analysis module of this platform uses a sentiment lexicon for sentiment score calculation. For details, please refer to: https://spssau.com/helps/textanalysis/sentiment.html.

4 According to the platform’s usage instructions, when performing sentiment analysis by line, SPSSAU defaults to compressing the sentiment score between −1 and 1, and determines the sentiment polarity based on the calculated sentiment score. The platform’s sentiment scores are continuous values obtained through an algorithm that comprehensively calculates the intensity of sentiment words in the text and accounts for modifiers (such as negation words and degree adverbs), followed by normalization. Within this range, negative values indicate a negative sentiment tendency, while positive values indicate a positive sentiment tendency. The closer the absolute value is to 0, the weaker the sentiment polarity and the more neutral the expression. The emotional polarity of sentiment keywords involves a degree of “flexible” (subjective) judgment; as scores approach the neutral zone, the distinction between positive and negative directions becomes less pronounced. Therefore, for setting sentiment classification thresholds, this study—based on the distribution characteristics of sentiment intensity along the continuum—narrows the absolute value of intervals near zero. This approach helps capture subtle emotional nuances more precisely and better distinguishes between “moderately positive” and “moderately negative” expressions. According to these thresholds, sentiment scores are categorized into four classes: strongly negative [−1, −1/3), moderately negative [−1/3, 0), moderately positive [0, 1/3), and strongly positive [1/3, 1]. For details, please refer to: https://spssau.com/helps/textanalysis/sentiment.html.

5 The workshop was recorded in its entirety with cameras set up in front of the participants after they signed the informed consent form. In principle, the recording of each session began when the first participant entered and ended when the last participant left. However, the event included a segment at the end where the participants selected bookmark gifts; this segment was not recorded on camera and was documented by staff recalling the participants’ reactions. A total of 11 events were recorded with 81 participating individuals. Excluding those whose facial expressions and body language could not be fully captured due to seating angles, those who did not participate throughout, and those who wore masks concealing their faces, 36 participants remained.

6 For example, Electromyographic (EMG) Biofeedback (De Witte et al., 2019), Electrodermal Activity (EDA) Biofeedback/Galvanic Skin Response (GSR) Biofeedback (Pop-Jordanova and Pop-Jordanov, 2020), Electroencephalographic (EEG) Biofeedback (Markiewcz, 2017), Skin Temperature Biofeedback (Credidio, 1982).

## References

[ref1] Allison S. T. Goethals G. R. (2014). ““Now he belongs to the ages”: the heroic leadership dynamic and deep narratives of greatness” in Conceptions of leadership: enduring ideas and emerging insights. eds. Goethals G. R. Allison S. T. Kramer R. M. Messick D. M. (New York, NY: Palgrave Macmillan), 167–183.

[ref2] Allison S. T. Goethals G. R. (2016). Hero worship: the elevation of the human spirit. J. Theory Soc. Behav. 46, 187–210. doi: 10.1111/jtsb.12094

[ref3] American Alliance of Museums (2013). Museums on call: How museums are addressing health issues. Arlington: American Alliance of Museums.

[ref4] Ander E. Thomson L. Noble G. Lanceley A. Menon U. Chatterjee H. (2011). Generic well-being outcomes: towards a conceptual framework for well-being outcomes in museums. Mus. Manag. Curatorship 26, 237–259. doi: 10.1080/09647775.2011.585798

[ref5] Anderson D. (1989). Learning history in museums. Mus. Manag. Curatorship 8, 357–368. doi: 10.1016/0260-4779(89)90003-4

[ref6] Andrews G. Issakidis C. Carter G. (2001). Shortfall in mental health service utilisation. Br. J. Psychiatry 179, 417–425. doi: 10.1192/bjp.179.5.417, 11689399

[ref7] Anh P. P. Tin N. T. (2021). The meaning of the lotus’s symbol in Bubbhist philosophy for Vietnam now. Turk. J. Comput. Math. Educ. 12, 6794–6807. doi: 10.17762/turcomat.v12i13.10054

[ref8] Annamary K. (2016). Colour preference to emotions in relation to the anxiety level among school children in Puducherry–a cross-sectional study. J. Clin. Diagn. Res. 10, ZC26–ZC30. doi: 10.7860/jcdr/2016/18506.8128, 27630948 PMC5020302

[ref9] Baddeley G. Evans L. Lajeunesse M. Legari S. (2017). Body talk: examining a collaborative multiple-visit program for visitors with eating disorders. J. Mus. Educ. 42, 345–353. doi: 10.1080/10598650.2017.1379278

[ref10] Bailey C. (2009). Embracing the icon: the feminist potential of the trans bodhisattva, Kuan Yin. Hypatia 24, 178–196. doi: 10.1111/j.1527-2001.2009.01051.x

[ref11] Bertamini M. Makin A. Rampone G. (2013). Implicit association of symmetry with positive valence, high arousal and simplicity. i-Perception 4, 317–327. doi: 10.1068/i0601jw

[ref12] Binnie J. (2010). Does viewing art in the museum reduce anxiety and improve wellbeing? Museums & Social Issues 5, 191–201. doi: 10.1179/msi.2010.5.2.191

[ref13] Blazhenkova O. Kumar M. M. (2018). Angular versus curved shapes: correspondences and emotional processing. Perception 47, 67–89. doi: 10.1177/0301006617731048, 28927319

[ref14] Bolen J. S. (1984). Goddesses in everywoman: a new psychology of women. New York, NY: Harper Colophon.

[ref15] Bondil N. Legari S. (2022). ““Museotherapy”: a new concept for promoting health, well-being, and therapy through art” in The Oxford handbook of the positive humanities. eds. Tay L. Pawelski J. O. (London: Oxford Academic), 483–499.

[ref16] Bruner J. (1986). Actual minds, possible worlds. Cambridge: Harvard University Press.

[ref17] Burkert W. (1996). Creation of the sacred: tracks of biology in early religions. Cambridge: Harvard University Press.

[ref18] Ceccarelli S. Cesta A. Cortellessa G. De Benedictis R. Fracasso F. Leopardi L. . (2024). Evaluating visitors’ experience in museum: comparing artificial intelligence and multi-partitioned analysis. Digit. Appl. Archaeol. Cult. Herit. 33:e00340. doi: 10.1016/j.daach.2024.e00340

[ref19] Chatterjee H. J. Camic P. M. (2015). The health and well-being potential of museums and art galleries. Arts Health 7, 183–186. doi: 10.1080/17533015.2015.1065594

[ref20] Chatterjee H. Vreeland S. Noble G. (2009). Museopathy: exploring the healing potential of handling museum objects. Museum Soc 7, 164–177. doi: 10.29311/mas.v7i3.145

[ref21] China National Narcotic Drugs Association (2023) The blue paper on China's mental health in 2023 released. Available online at: https://mp.weixin.qq.com/s/CJS1FY5-yHKn2l-YXdmLRQ (Accessed November 30, 2024).

[ref22] Clauw D. J. Häuser W. Cohen S. P. Fitzcharles M. A. (2020). Considering the potential for an increase in chronic pain after the COVID-19 pandemic. Pain 161, 1694–1697. doi: 10.1097/j.pain.0000000000001950, 32701829 PMC7302093

[ref23] Clobert M. (2021). East versus west: psychology of religion in east Asian cultures. Curr. Opin. Psychol. 40, 61–66. doi: 10.1016/j.copsyc.2020.08.021, 33022518

[ref24] Clow A. Fredhoi C. (2006). Normalisation of salivary cortisol levels and self-report stress by a brief lunchtime visit to an art gallery by London city workers. J. Holist. Healthc. 3, 29–32.

[ref25] Coles A. Harrison F. Todd S. (2019). Flexing the frame: therapist experiences of museum-based group art psychotherapy for adults with complex mental health difficulties. Int. J. Art Ther. 24, 56–67. doi: 10.1080/17454832.2018.1564346

[ref26] Credidio S. G. (1982). Comparative effectiveness of patterned biofeedback vs. meditation training on EMG and skin temperature changes. Behav. Res. Ther. 20, 233–241. doi: 10.1016/0005-7967(82)90141-3, 7046724

[ref27] Cull R. Cull D. (2022). Museums and well-being. London: Routledge.

[ref28] Cutler D. (2022). Creatively minded at the museum: creative and mental health activity in museums. London: The Baring Foundation.

[ref29] Daniali H. Martinussen M. Flaten M. A. (2023). A global meta-analysis of depression, anxiety, and stress before and during COVID-19. Health Psychol. 42, 124–138. doi: 10.1037/hea0001259, 36802363

[ref30] De Jong S. (2018). Sentimental education. Sound and silence at history museums. Mus. Soc. 16, 88–106. doi: 10.29311/mas.v16i1.2537

[ref31] De Witte N. A. J. Buyck I. Van Daele T. (2019). Combining biofeedback with stress management interventions: a systematic review of physiological and psychological effects. Appl. Psychophysiol. Biofeedback 44, 71–82. doi: 10.1007/s10484-018-09427-7, 30604099

[ref32] Deane K. Carman M. Fitch M. (2000). The cancer journey: bridging art therapy and museum education. Can. Oncol. Nurs. J. 10, 140–142. doi: 10.5737/1181912x104140142, 11894320

[ref33] Dejkameh M. Candiano J. Shipps R. (2018) Paving new ways to exploration in cultural institutions: a gallery guide to inclusive art-based engagement in cultural institutions. Queens museum of art. Available online at: https://queensmuseum.org/wp-content/uploads/2022/08/PAVE-Guide_web.pdf (Accessed November 30, 2024).

[ref34] Dejkameh M. R. Shipps R. (2019). From please touch to art access: the expansion of a museum-based art therapy program. Art Ther. 35, 211–217. doi: 10.1080/07421656.2018.1540821

[ref35] Dierking L. D. Falk J. H. (1992). Redefining the museum experience: the interactive experience model. Visit. Stud. 4, 173–176.

[ref36] Du J. (2020). Xishengyou tiaojie yiyuzheng huanzhe fuxing renzhi pianxiang de shenjing jizhi yanjiu [喜胜忧调节抑郁症患者负性认知偏向的神经机制研究] [a study on the neural mechanism of "delighting adjusting sorrow" in modulating negative cognitive bias in depressed patients]. [doctoral thesis]. China: Beijing University of Chinese Medicine.

[ref37] Ekman P. Friesen W. V. (1978). Facial action coding system. Palo Alto: Consulting Psychologists Press.

[ref38] Ekman P. Friesen W. V. Ellsworth P. (1972). Emotion in the human face: Guide-lines for research and an integration of findings. New York, NY: Pergamon Press.

[ref39] Ellsworth P. C. (2024). “Appraisal theories of emotions” in Emotion theory: the Routledge comprehensive guide. ed. Scarantino A. (New York, NY: Routledge), 331–349.

[ref40] Falk J. H. Dierking L. D. (2016). The museum experience revisited. London: Routledge doi: 10.4324/9781315417851.

[ref41] Ferilli G. Grossi E. Sacco P. L. Tavano Blessi G. (2016). Museum environments, visitors’ behaviour, and well-being: beyond the conventional wisdom. Mus. Manag. Curat. 32, 80–102. doi: 10.1080/09647775.2016.1239125

[ref42] Fujiwara D. (2013) Museums and happiness: the value of participating in museums and the arts. Available online at: http://www.happymuseumproject.org/wp-content/uploads/2013/04/Museums_and_happiness_DFujiwara_April2013.pdf (Accessed November 30, 2024).

[ref43] Geratowski L. (2020). Visual anxiolytics: Developing theory and design guidelines for abstract affective visualizations aimed at alleviating episodes of anxiety. [master of Arts thesis]. Finland: Aalto University.

[ref44] Ghadim M. R. Daugherty L. (2021). Museum-based art therapy: a collaborative effort with access, education, and public programs. London: Routledge.

[ref45] Goodman R. F. Fahnestock A. H. (2002). The day our world changed: children's art of 9/11. New York, NY: Harry N. Abrams.

[ref46] Gregory K. Witcomb A. (2007). “Museum revolutions: how museums change and are changed” in Beyond nostalgia: the role of affect in generating historical understanding at heritage sites. eds. Knell S. MacLeod S. Watson S. (London: Routledge), 263–275.

[ref47] Greimas A. J. Courtes J. Rengstorf M. (1976). The cognitive dimension of narrative discourse. New Lit. Hist. 7, 433–447. doi: 10.2307/468554

[ref48] Hartman A. (2019). Museum as a space for therapeutic art experiences for adolescents with high functioning autism (HFA). [doctor of Philosophy]. Florida: Florida State University.

[ref49] Hepper E. G. Ritchie T. D. Sedikides C. Wildschut T. (2012). Odyssey's end: lay conceptions of nostalgia reflect its original homeric meaning. Emotion 12, 102–119. doi: 10.1037/a0025167, 21859192

[ref50] Huang Q. Balsys R.J. (2009) Applying fractal and Chaos theory to animation in the Chinese literati tradition, in 2009 Sixth International Conference on Computer Graphics, Imaging and Visualization Tianjin: IEEE, 112–122.

[ref51] Innes M. Sharp H. (2021). Historical empathy and museum culture. J. Mus. Educ. 46, 307–320. doi: 10.1080/10598650.2021.1954771

[ref52] Ioannides E. (2016). Museums as therapeutic environments and the contribution of art therapy. Mus. Int. 68, 98–109. doi: 10.1111/muse.12125

[ref53] Jiang D. (2004). Dongwu tuteng chongbai [动物 图腾 崇拜] [Animal, totem, worship]. J. Dalian Minzu Univ. 2, 6–10.

[ref54] Jiang Q. Ma L. Yue M. (2022). Animation narrative on stress relief and psychological cognitive development in adolescents. Occup. Ther. Int. 2022, 1–10. doi: 10.1155/2022/1111488, 36101670 PMC9463020

[ref55] Kaiser P. K. (1984). Physiological response to color: a critical review. Color. Res. Appl. 9, 29–36. doi: 10.1002/col.5080090106

[ref56] Kaplan S. (1995). The restorative benefits of nature: toward an integrative framework. J. Environ. Psychol. 15, 169–182. doi: 10.1016/0272-4944(95)90001-2

[ref57] Kılıçarslan Ö. Yozukmaz N. Albayrak T. Buhalis D. (2024). The impacts of Metaverse on tourist behaviour and marketing implications. Curr. Issues Tour. 28, 622–642. doi: 10.1080/13683500.2024.2326989

[ref58] Kim N. K. (2020). Symbolism of snakes that stimulate restoration and transformation of visceral vitality. J. Symb. Sandplay Therapy 11, 191–230. doi: 10.12964/jsst.20005

[ref59] Koole S. L. Sin M. T. A. Schneider I. K. (2013). Embodied terror management: interpersonal touch alleviates existential concerns among individuals with low self-esteem. Psychol. Sci. 25, 30–37. doi: 10.1177/0956797613483478, 24190907

[ref60] Koster E. H. Baumann S. H. (2005). “Liberty science center in the United States: a mission focused on external relevance” in Looking reality in the eye: museums and social responsibility. eds. Janes R. R. Conaty G. T. (Calgary: University of Calgary Press and Museums Association of Saskatchewan), 85–112.

[ref61] Kowen M. R. (2019). A symbol of healing with feminine vitality: the willow. J. Symb. Sandplay Ther. 10, 41–73. doi: 10.12964/jsst.19003

[ref62] Lanceley A. Noble G. Johnson M. Balogun N. Chatterjee H. Menon U. (2012). Investigating the therapeutic potential of a heritage-object focused intervention: a qualitative study. J. Health Psychol. 17, 809–820. doi: 10.1177/1359105311426625, 22104664

[ref63] Lazarus R. (2021). Individual differences in emotion, the nature of emotion: fundamental questions. New York, NY: Oxford University Press.

[ref64] LeDoux J. E. (2022). “Emotional processing, but not emotions, can occur unconsciously” in The nature of emotion: fundamental questions. eds. Ekman P. Davidson R. (New York, NY: Oxford University Press), 291–292.

[ref65] Li Q. Li J. Fan Y. (2025). Addressing mental health in university students: a call for action. Front. Public Health 13:1614999. doi: 10.3389/fpubh.2025.1614999, 40606073 PMC12213389

[ref66] Li Y. Pan Q. Yang T. Wang S. Tang J. Cambria E. (2017). Learning word representations for sentiment analysis. Cogn. Comput. 9, 843–851. doi: 10.1007/s12559-017-9492-2

[ref67] Li S. H. Zhang W. X. (2009). Yuanyi liaofa kexue yanjiu jinzhan 园艺疗法科学研究进展 (Progress in horticultural therapy scientific research). Chin. Landsc. Archit. 8, 19–23.

[ref68] Luebke J. F. Watters J. V. Packer J. Miller L. J. Powell D. M. (2016). Zoo visitors' affective responses to observing animal behaviors. Vis. Stud. 19, 60–76. doi: 10.1080/10645578.2016.1144028

[ref69] Mangione G. (2018). The art and nature of health: a study of therapeutic practice in museums. Sociol. Health Illn. 40, 283–296. doi: 10.1111/1467-9566.12618, 29464772

[ref70] Markiewcz R. (2017). The use of EEG biofeedback/neurofeedback in psychiatric rehabilitation. Zastosowanie EEG biofeedback/neurofeedback w rehabilitacji psychiatrycznej. Psychiatr. Pol. 51, 1095–1106. doi: 10.12740/PP/68919, 29432505

[ref71] Melton A. W. (1972). Visitor behavior in museums: some early research in environmental design. Hum. Factors 14, 393–403. doi: 10.1177/001872087201400503

[ref72] Moors A. (2014). Flavors of appraisal theories of emotion. Emot. Rev. 6, 303–307. doi: 10.1177/1754073914534477

[ref73] Nimer J. Lundahl B. (2007). Animal-assisted therapy: a meta-analysis. Anthrozoös 20, 225–238. doi: 10.2752/089279307x224773

[ref74] Olfson M. Klerman G. L. (1992). Depressive symptoms and mental health service utilization in a community sample. Soc. Psychiatry Psychiatr. Epidemiol. 27, 161–167. doi: 10.1007/bf00789000, 1411743

[ref75] Packer J. Ballantyne R. (2016). Conceptualizing the visitor experience: a review of literature and development of a multifaceted model. Vis. Stud. 19, 128–143. doi: 10.1080/10645578.2016.1144023

[ref76] Palmer S. E. Schloss K. B. Sammartino J. (2013). Visual aesthetics and human preference. Annu. Rev. Psychol. 64, 77–107. doi: 10.1146/annurev-psych-120710-100504, 23020642

[ref77] Pasqualotto A. Ng M. Tan Z. Y. Kitada R. (2020). Tactile perception of pleasantness in relation to perceived softness. Sci. Rep. 10:11189. doi: 10.1038/s41598-020-68034-x, 32636415 PMC7341757

[ref78] Pearce P. (2009). The relationship between positive psychology and tourist behavior studies. Tour. Anal. 14, 37–48. doi: 10.3727/108354209788970153

[ref79] Pop-Jordanova N. Pop-Jordanov J. (2020). Electrodermal activity and stress assessment. Prilozi Makedonska akademija na naukite i umetnostite. Oddelenie za medicinski nauki 41, 5–15. doi: 10.2478/prilozi-2020-0028, 33011695

[ref80] Qin Z. Song Y. (2020). The sacred power of beauty: examining the perceptual effect of Buddhist symbols on happiness and life satisfaction in China. Int. J. Environ. Res. Public Health 17:2551. doi: 10.3390/ijerph17072551, 32276426 PMC7177423

[ref81] Reed N. C. L. (2018). Understanding visitor happiness in museums. Washington: University of Washington.

[ref82] Rhodes G. (2006). The evolutionary psychology of facial beauty. Annu. Rev. Psychol. 57, 199–226. doi: 10.1146/annurev.psych.57.102904.190208, 16318594

[ref83] Robinson E. (1928). The behaviour of the museum visitor. New Series No. 5. Washington, DC: American Association of Museums.

[ref84] Rosenblatt B. (2014). Museum education and art therapy: promoting wellness in older adults. J. Mus. Educ. 39, 293–301. doi: 10.1080/10598650.2014.11510821

[ref85] Salgado-Montejo A. Alvarado J. A. Velasco C. Salgado C. J. Hasse K. Spence C. (2015). The sweetest thing: the influence of angularity, symmetry, and the number of elements on shape-valence and shape-taste matches. Front. Psychol. 6:1382. doi: 10.3389/fpsyg.2015.01382, 26441757 PMC4569812

[ref86] Santomauro D. F. Herrera A. M. M. Shadid J. Zheng P. Ashbaugh C. Pigott D. M. . (2021). Global prevalence and burden of depressive and anxiety disorders in 204 countries and territories in 2020 due to the COVID-19 pandemic. Lancet 398, 1700–1712. doi: 10.1016/s0140-6736(21)02143-7 34634250 PMC8500697

[ref87] Schauss A. G. (1979). Tranquilizing effect of color reduces aggressive behavior and potential violence. J. Orthomol. Psychiatry 8, 218–221.

[ref88] Serenko A. Turel O. (2019). A dual-attitude model of system use: the effect of explicit and implicit attitudes. Inf. Manag. 56, 657–668. doi: 10.1016/j.im.2018.10.009

[ref89] Sheih C. S. M. (2014). The emotional healing efficacy of romance fiction for undergraduates with love-related emotional disturbance problems: an exploratory research. J. Libr. Inf. Stud. 12, 39–79. doi: 10.6182/jlis.2014.12(2).039

[ref90] Shevchuk N. A. (2008). Adapted cold shower as a potential treatment for depression. Med. Hypotheses 70, 995–1001. doi: 10.1016/j.mehy.2007.04.052, 17993252

[ref91] Smith L. (2020). “Uses of heritage” in Encyclopedia of global archaeology. ed. Smith C. (Cham: Springer International Publishing), 10969–10974.

[ref92] Smith L. Campbell G. (2016). “The elephant in the room: heritage, affect, and emotions” in A companion to heritage studies. eds. Logan W. Craith M. N. Kockel U. (New York, NY: Wiley), 443–460.

[ref93] Sone Y. Harada T. Miyamoto M. Fukumoto Y. Tani N. Shintani A. . (2007). The effect of group reminiscence in nostalgic room for mildly demented elderly: evaluation with NIkS during dementia assessment test. J. Physiol. Anthropol. 26:613.

[ref94] Sontag S. (2011). Illness as metaphor and aids and its metaphors. New York, NY: Macmillan.

[ref95] Soranzo A. Petrelli D. Ciolfi L. Reidy J. (2018). On the perceptual aesthetics of interactive objects. Q. J. Exp. Psychol. 71, 2586–2602. doi: 10.1177/1747021817749228, 29364061 PMC6293455

[ref96] Spielberger C. D. (1989). State-trait anxiety inventory: bibliography. 2nd Edn. Palo Alto: Consulting Psychologists Press.

[ref97] Taylor R. P. (2006). Reduction of physiological stress using fractal art and architecture. Leonardo 39, 245–251. doi: 10.1162/leon.2006.39.3.245

[ref98] Tolia-Kelly D. P. Waterton E. Watson S. (2016). Heritage, affect and emotion: politics, practices and infrastructures. New York, NY: Routledge.

[ref99] Valdez P. Mehrabian A. (1994). Effects of color on emotions. J. Exp. Psychol. Gen. 123, 394–409. doi: 10.1037//0096-3445.123.4.394, 7996122

[ref100] Van Horen F. Mussweiler T. (2014). Soft assurance: coping with uncertainty through haptic sensations. J. Exp. Soc. Psychol. 54, 73–80. doi: 10.1016/j.jesp.2014.04.008 PMC4692610 26436729

[ref101] Vassilieva J. (2016). “The narrative turn in psychology” in Narrative psychology (London: Palgrave Macmillan), 9–47.

[ref102] Voss R. F. Wyatt J. C. Y. (1993). “Multifractals and the local connected fractal dimension” in Applications of fractals and Chaos. eds. Crilly A. J. Earnshaw R. A. Jones H. (Berlin: Springer), 171–192.

[ref103] Wang H. (2015). Chanhou yiyuzhe yingyong qingzhixiangsheng xinli ganyu de xiaoguo fenxi 产后抑郁者应用情志相胜心理干预的效果分析 (Analysis of the effect of applying psychological interventions for postpartum depressed people with emotional and psychological philanthropy). J. Nurs. 22, 71–74.

[ref104] Wang X. Liao F. Yuan W. Wang Y. (2025). 10 the therapeutic effect of immersive digital museum viewing experience mode on relieving psychological anxiety of college students. Curr. Opin. Psychiatry 38:e3. doi: 10.1097/01.yco.0001119128.73200.d1

[ref105] Wang S. Wu X. (2022). Revolutionary exhibition and youth identity: a visitor study of the Shanghai Sihang warehouse battle memorial. Mus. Manag. Curatorsh. 38, 293–316. doi: 10.1080/09647775.2022.2158909, 41307611

[ref106] Wankhade M. Rao A. C. S. Kulkarni C. (2022). A survey on sentiment analysis methods, applications, and challenges. Artif. Intell. Rev. 55, 5731–5780. doi: 10.1007/s10462-022-10144-1

[ref107] Wathelet M. Fovet T. Jousset A. Duhem S. Habran E. Horn M. . (2021). Prevalence of and factors associated with post-traumatic stress disorder among French university students 1 month after the COVID-19 lockdown. Transl. Psychiatry 11:327. doi: 10.1038/s41398-021-01438-z, 34045442 PMC8157529

[ref108] Watson S. (2015). “Emotions in the history museum” in The international handbooks of museum studies: museum and theory. eds. Witcomb A. Message K. (New York, NY: Wiley), 259–484.

[ref109] Watson S. (2016). “Why do emotions matter in museums and heritage sites” in Sensitive pasts: questioning heritage in education. eds. Boxtel C. V. Grever M. Klein S. (New York, NY: Berghahn Books), 75–91.

[ref110] Watson E. Coles A. Jury H. (2021). ‘A space that worked for them’: museum-based art psychotherapy, power dynamics, social inclusion and autonomy. Int. J. Art Ther. 26, 137–146. doi: 10.1080/17454832.2020.1866046

[ref111] Wells V. K. (2014). Behavioural psychology, marketing and consumer behaviour: a literature review and future research agenda. J. Mark. Manag. 30, 1119–1158. doi: 10.1080/0267257X.2014.929161

[ref112] White M. (2024). Maps of narrative practice. New York, NY: WW Norton & Company.

[ref113] Witcomb A. (2014). Look, listen and feel’: the first peoples exhibition at the Bunjilaka gallery, Melbourne museum. Thema Rev. Mus. Civilistion 1, 49–62. doi: 10.3316/informit.352117074472071

[ref114] Wu T. Jia X. Shi H. Niu J. Yin X. Xie J. . (2021). Prevalence of mental health problems during the COVID-19 pandemic: a systematic review and meta-analysis. J. Affect. Disord. 281, 91–98. doi: 10.1016/j.jad.2020.11.117, 33310451 PMC7710473

[ref115] Xie Q. Yang Q. Wang Z. (2022). Zhongyi feiyaowu liaofa zhiliao yiyuzheng de yanjiu 中医非药物疗法治疗抑郁症的研究 (A study on nonpharmacological treatment of depression in Chinese medicine). J. Basic Chin. Med. 28, 491–494.

[ref116] Yim S. H. (2022). Cultural heritage through the lens of community psychology and narrative therapy: a community project on Chinese and Vietnamese diaspora in London. Int. J. Herit. Stud. 28, 970–983. doi: 10.1080/13527258.2022.2108486

[ref117] Zhang H. Lyu R. Zheng Z. Chen K. Li Q. Wei S. . (2017). Qingzhixiangsheng liaofa zai yiyuzheng zhong yingyong jiqi jizhi yanjiu 情志相胜疗法在抑郁症中应用及其机制研究 (Research progress of emotional therapy in the treatment of depression). J. Liaoning Univ. Tradit. Chin. Med. 19, 70–73.

[ref118] Zhou S. Chen S. Li S. (2021). The shape effect: round shapes increase consumers' preference for hedonic foods. Psychol. Mark. 38, 2051–2072. doi: 10.1002/mar.21547

[ref119] Zhou X. Sedikides C. Wildschut T. Gao D. G. (2008). Counteracting loneliness: on the restorative function of nostalgia. Psychol. Sci. 19, 1023–1029. doi: 10.1111/j.1467-9280.2008.02194.x, 19000213

